# Integrating network analysis and machine learning to explore the pharmacological targets and associations of oleanolic acid in lung adenocarcinoma

**DOI:** 10.3389/fphar.2026.1841232

**Published:** 2026-09-18

**Authors:** Ying Zeng, Hongting Jiang, Fei Zhang, Cha Luo, Zhonglian Wang, Zhiming Ren, Zhihao Xu, Wen Li, Qing Ye, Wei Jian, Jing Zhang, Qiaofen Fu

**Affiliations:** Department of Radiation Oncology, The First Affiliated Hospital of Kunming Medical University, Kunming, Yunnan, China

**Keywords:** biomarker, lung adenocarcinoma, machine learning, network analysis, oleanolic acid

## Abstract

**Background:**

Lung adenocarcinoma (LUAD), a major type of non-small cell lung cancer, has high incidence and mortality rates. Oleanolic acid (OA), a natural pentacyclic triterpene compound, has demonstrated potential anti-tumor effects but its pharmacological effects in LUAD remain unclear.

**Purpose:**

This study aims to identify the potential targets and action pathways of OA in LUAD using network analysis, transcriptomics, molecular docking, animal experiments, and preliminary clinical sample assessment.

**Methods:**

Potential candidate genes for OA in LUAD were identified from public databases including PubChem, GeneCards, and TCGA. GO and KEGG enrichment analyses were performed using Metascape, and a PPI network was constructed. Machine learning methods (Lasso regression and SVM-RFE) were employed to refine the candidate genes and identify key biomarkers. Prognostic, clinicopathological, GSEA, mutation, and immune infiltration analyses were conducted. Molecular docking was performed to estimate the theoretical binding affinity between OA and the biomarkers, which was further evaluated using animal models and clinical samples.

**Results:**

21 candidate genes were initially identified, with TOP2A and ALOX5AP emerging as key biomarkers. High TOP2A expression and low ALOX5AP expression were associated with poor prognosis. GSEA showed co-enrichment in the IgA production pathway. Mutation analysis indicated higher amplification propensity for TOP2A. Both biomarkers correlated with immune cells. Molecular docking and animal experiments suggested OA’s regulatory effects on these genes, with clinical sample evaluation.

**Conclusion:**

Utilizing computational screening as a theoretical starting point alongside preliminary experimental evaluations, this study identifies ALOX5AP and TOP2A as potential pharmacological biomarkers associated with OA treatment in LUAD. Rather than asserting definitive specific targeting, these findings provide preliminary associative evidence. Although direct causal relationships require further functional investigation, this work establishes a pharmacological and theoretical foundation for understanding OA’s anti-tumor effects and future therapeutic potential.

## 1 Introduction

Lung adenocarcinoma (LUAD) is the most common subtype of non-small cell lung cancer (NSCLC) and poses a severe threat to global human health (Duan et al., 2023). Despite advancements in targeted therapies and immune checkpoint inhibitors (Liao et al., 2023), clinical benefits remain highly limited due to drug resistance and low response rates (Hao et al., 2022; Liu et al., 2023). Therefore, exploring novel therapeutic agents and their underlying molecular targets for LUAD remains extremely crucial (Lim and Ma, 2019). In recent years, integrated bioinformatics workflows combining omics data analysis, network modeling, and machine learning have been widely adopted and proven highly effective in elucidating complex molecular mechanisms in lung cancer (Andalib et al., 2024). While such computational pipelines have become standard practice in pharmacological research, their true scientific value lies in their capacity to generate robust, testable hypotheses for specific compounds.

Oleanolic acid (OA, 3-β-3-Hydroxyolean-12-en-28-oic acid) is a pentacyclic triterpenoid widely distributed in numerous traditional medicinal plants, including Ligustrum lucidum, Forsythia suspensa, and Panax ginseng (Subramanya et al., 2018). Historically, botanical decoctions rich in OA have been extensively utilized in traditional medicine systems to treat inflammatory conditions and hepatic disorders. Building upon this ethnopharmacological foundation, modern pharmacological studies have demonstrated that OA possesses diverse biological activities and exhibits broad-spectrum anti-tumor effects (Huang et al., 2022; Castrejón-Jiménez et al., 2019; Abulaiti et al., 2024; Deng et al., 2024). Although a substantial body of literature has established the basic anti-tumor phenotypic effects of OA across various cancers, including lung cancer (e.g., inducing apoptosis and cell cycle arrest) (Zhao et al., 2015), previous investigations have predominantly focused on broad macroscopic observations. The precise systemic molecular targets and the specific modulatory mechanisms of OA within the tumor immune microenvironment (TIME) remain a critical knowledge gap. Unlike prior observational studies, the present research integrates machine learning with multi-omics approaches to systematically identify TOP2A and ALOX5AP as novel dual-synergistic targets of OA in LUAD. By delineating these specific regulatory axes, this study transcends traditional phenotypic descriptions and provides a deeper molecular foundation for targeted interventions.

Against this background, a central hypothesis is proposed: OA exerts its pharmacological effects in LUAD by modulating specific molecular targets and the tumor microenvironment network. To formally test this hypothesis, rather than merely seeking to validate computational predictions, the present study employs network analysis and transcriptomics to generate an initial pool of candidate targets. This is subsequently followed by empirical testing and assessment using animal models and clinical sample analysis. Ultimately, this approach aims to address the fundamental research question of how OA acts at the molecular level in LUAD and to critically evaluate the translational potential of these computationally derived targets.

## 2 Materials and methods

### 2.1 Systematic screening of candidate genes and testing cohort construction

This study employed an integrated bioinformatics approach to systematically identify potential therapeutic targets of OA in LUAD. The complete structural information and canonical SMILES identifier (CC1(CCC2(CCC3(C (=CCC4C3(CCC5C4(CCC(C5(C)C)O)C)C)C2C1)C)C (=O)O)C) for OA were obtained from the PubChem database. Potential OA target genes were subsequently predicted using the SwissTargetPrediction database and designated as the Swiss Target set.

LUAD-associated genes were compiled through comprehensive searches of GeneCards, Online Mendelian Inheritance in Man (OMIM), and DisGeNET databases using “lung adenocarcinoma” as the search term. These genes were organized into two complementary datasets: the GeneCard set (integrating GeneCards and OMIM data) and the DisGene set (representing DisGeNET data exclusively).

Transcriptomic data were obtained from The Cancer Genome Atlas (TCGA) database, comprising 524 tumor and 59 normal tissue samples as the training set. Differential expression analysis was performed using DESeq2 (v1.42.0) with thresholds of |log2 fold change|>0.5 and adjusted p-value<0.05. An initial permissive screening threshold of |log2 fold change| > 0.5 and adjusted p-value <0.05 was adopted based on previous exploratory transcriptomic studies to minimize false negatives at the preliminary stage. Importantly, a retrospective sensitivity analysis confirmed that the core biomarkers ultimately identified by the downstream machine learning pipeline far exceeded stricter conventional thresholds (i.e., |log2FC| > 1.0), thereby guaranteeing the statistical robustness of the final targets. Results were visualized through volcano plots (ggplot2 v3.4.1) and heatmaps (pheatmap v1.0.12).

To ensure data reliability, highly authoritative and complementary public databases were deliberately selected for initial target prediction. Specifically, PubChem (maintained by the NIH) was utilized for its standardized chemical structure validation; GeneCards was employed as it represents a highly integrated repository of multi-dimensional human gene data; and DisGeNET was selected for its focus on high-quality, evidence-based gene-disease associations (incorporating GWAS, ClinVar, and literature text-mining). To strictly handle potential data redundancy and eliminate low-confidence, database-specific artifacts, the candidate genes were not simply merged. Instead, an initial, exploratory pool of candidate genes was generated by computing the strict mathematical intersection of four distinct data sources (SwissTargetPrediction, GeneCards, DisGeNET, and TCGA DEGs). This intersection strategy ensured that only genes supported by multiple independent lines of evidence were retained for downstream analysis.

An initial, exploratory pool of candidate genes potentially associated with OA in LUAD was generated by cross-referencing four distinct gene sets including Swiss Target, GeneCard, DisGene, and DEGs. The GSE140797 (GPL13497) dataset was retrieved from the NCBI GEO database as an independent evaluation cohort, comprising seven LUAD tissues and their matched adjacent non-tumor controls, and was employed for both differential-expression analysis and ROC-curve assessment. For TCGA data preprocessing, RNA-seq raw count data, alongside corresponding clinical and survival information for LUAD, were downloaded. Samples were strictly filtered to retain only primary tumor and adjacent normal tissues. Ensembl IDs were converted to gene symbols based on the GRCh37.87 annotation, retaining exclusively protein-coding genes and removing duplicates to construct the expression matrix. Raw counts were integer-processed and subjected to variance stabilizing transformation (VST) normalization using DESeq2 prior to differential expression analysis. For the independent GSE140797, microarray data were retrieved using the GEOquery package. Following phenotypic grouping, data quality was assessed via boxplots, and cross-sample normalization was performed utilizing the limma package. Probe IDs were mapped to gene symbols using the GPL13497 annotation, yielding the final gene-level expression matrix.

### 2.2 Enrichment analysis of candidate genes

Functional enrichment analysis was subsequently performed on the 21 candidate genes to elucidate their biological significance. Gene Ontology (GO) and Kyoto Encyclopedia of Genes and Genomes (KEGG) pathway analyses were conducted using the ClusterProfiler package (v 3.18.1) (Yu et al., 2012) with a significance threshold of p < 0.05. Additionally, protein-protein interaction (PPI) networks were constructed for the candidate genes through the STRING database (https://string-db.org/) using an interaction score threshold>0.4 (He et al., 2020). The primary aim of the PPI analysis was to identify potential core regulatory genes among the candidates by mapping their interaction landscape, rather than to perform further gene screening.

### 2.3 Screening and evaluation of candidate biomarkers

Candidate biomarkers were screened using the Least Absolute Shrinkage and Selection Operator (LASSO) and the Support Vector Machine Recursive Feature Elimination (SVM-RFE) algorithms. Lasso regression, developed by Tibshirani in 1996, is a linear regression technique that utilizes L1 regularization to shrink coefficients and select the variables most strongly correlated with the response (Zhang et al., 2021). SVM-RFE is an RFE application that uses weight magnitude for ranking and systematically removes the feature with the least influence on the SVM weight-vector magnitude (Gioia et al., 2022). In this study, we used the R package “glmnet” (version 4.1–4) to implement LASSO regression and the R package “e1071”(version 1.7–13) to implement the SVM-RFE algorithm. The intersection of the genes identified by both algorithms was defined as the candidate biomarkers. To mitigate the risk of overfitting inherent to high-dimensional transcriptomic data and to ensure the robustness and transparency of the gene selection strategy, strict 10-fold cross-validation (CV) was implemented for both machine learning algorithms. In the LASSO regression model, the regularization parameter (λ) was optimized via 10-fold CV. The parameter lambda. min, which yielded the minimum CV error, was selected as the optimal threshold to extract features with non-zero regression coefficients. For the SVM-RFE algorithm, a 10-fold CV framework was similarly applied to optimize feature ranking stability. A linear kernel was selected due to its suitability for high-dimensional feature classification and weight calculation. The cost parameter was set to 10 to appropriately balance the classification margin and misclassification error. To optimize computational efficiency and screening precision, the feature elimination strategy dynamically halved the feature set when the number of variables exceeded 100, and sequentially eliminated single features when the count fell below 100. Feature ranking was determined by the normalized standard deviation of weights across multiple CV iterations. Furthermore, grid search optimization was utilized to fine-tune SVM hyperparameters, selecting the feature set with the highest average rank and optimal classification performance.

To deeply explore the expression patterns of these candidate genes in LUAD, we used the Wilcoxon test to analyze the data in the training set and the testing cohort respectively, revealing the expression differences of the candidate genes in cancer tissues and adjacent tissues. The ability of candidate biomarkers to distinguish cancer from adjacent tissues was evaluated by constructing the receiver operating characteristic (ROC) curve, and the genes with area under the curve (AUC) values greater than 0.9 within TCGA-LUAD and GSE140797 were defined as biomarkers. Paired-sample analysis of the identified biomarkers was conducted in the TCGA-LUAD dataset, comparing tumor tissues with matched adjacent normal tissues. In the UALCAN database (http://ualcan.path.uab.edu/), the protein expression levels of the biomarker genes in LUAD tissues and normal tissues were retrieved. This was conducted to evaluate the consistency of the expression patterns of the biomarkers between the transcriptomic and proteomic levels.

### 2.4 Prognosis and clinical features analysis of biomarkers

In the TCGA-LUAD dataset, LUAD samples were divided into high expression group and low expression group according to the optimal threshold of key gene expression level. Then, the survminer software package (version 0.4.9) was used to generate Kaplan-Meier (KM) survival curves of patients in each group to evaluate the differences in survival outcomes (Liu et al., 2021). p < 0.05 was considered statistically significant.

Additionally, the Wilcoxon rank-sum test was employed to assess the differential expression levels of the two candidate genes across various clinical parameters, including age, gender, pathologic T stage, pathologic N stage, overall stage, and overall survival (OS). To quantify the prognostic impact of individual biomarkers, univariate Cox proportional hazards regression was first performed using the R package “survival” to calculate the Hazard Ratios (HR) and 95% Confidence Intervals (CI). Subsequently, to rigorously ascertain the independent prognostic value of the identified biomarkers, a multivariate Cox regression model was constructed. Initially, ALOX5AP expression, TOP2A expression, age, gender, and overall clinical stage were incorporated into the model as covariates. Prior to final model construction, the proportional hazards (PH) assumption was statistically evaluated for all variables using Schoenfeld residuals. Because the variable representing gender violated the PH assumption (p < 0.05), it was systematically excluded to maintain the statistical validity of the model. The refined multivariate Cox regression analysis ultimately included age, clinical stage, ALOX5AP, and TOP2A to calculate the adjusted HR and 95% CI, thereby identifying independent prognostic factors for overall survival in LUAD. This analysis aimed to explore potential associations between clinical characteristics and the expression of these key genes, with statistical significance set at p < 0.05.

### 2.5 Gene set enrichment analysis

To elucidate the biological mechanisms and pathways associated with the key genes, Gene Set Enrichment Analysis (GSEA) was performed. First, the analysis of differences between groups with high and low expression of the key genes was calculated, and the multiplicity of differences was ranked. h. all.v2023.1. Hs.symbols.gmt and c2. cp.kegg.v2023.1. Hs.symbols.gmt were utilized as background sets, then GSEA was performed by GSVA package (v 1.44.5) (Hä et al., 2013) (adj.p-value<0.05, |normalized enrichment score (NES)|>1).

### 2.6 Mutation analysis and immune microenvironment analysis

The Gene Set Cancer Analysis (GSCA, http://bioinfo.life.hust.edu.cn/GSCA/) database was a comprehensive resource for gene set analysis in cancer (Teng et al., 2022). The single-nucleotide variation (SNV), copy-number variation (CNV), and methylation profiles of biomarker genes in LUAD were systematically characterized using GSCA. Spearman correlations between biomarker expression and immune-cell fractions were computed with the psych package (v 2.2.9) (Robles-Jimenez et al., 2021). To quantify tumour-versus-normal immune infiltration, CIBERSORT was employed to estimate the relative abundances of 22 immune-cell populations in TCGA-LUAD samples (Newman et al., 2015). The result was visualized with a radar plot.

To further explore the association between the biomarker gene and immune cells, tumor samples from TCGA-LUAD (with samples lacking clinical information or duplicated samples excluded, ensuring each group contained ≥3 samples and no zero variance) were divided into high and low expression groups based on the median expression level of the marker gene. To ensure the robustness of the immune infiltration analysis and mitigate biases inherent to single algorithms, a complementary, multi-tiered approach utilizing ESTIMATE, CIBERSORT, and ssGSEA was explicitly justified and employed. First, ESTIMATE was utilized to calculate stromal and immune scores, providing a rapid and robust macro-level assessment of global tumor microenvironment (TME) purity and overall immune enrichment. Subsequently, CIBERSORT, a standard method employing support vector regression based on a validated gene signature matrix, was selected to finely delineate the relative proportions of 22 specific immune cell subtypes. Finally, ssGSEA was applied to quantify the enrichment levels of immune-related functional modules, thereby capturing the functional activation states and phenotypes of the immune cells rather than merely their abundance. Together, these three methodologies mutually validate and complement each other across three dimensions—global immune levels, specific cellular composition, and functional phenotypes—ensuring a highly robust, comprehensive profiling of the TME. Meanwhile, Spearman analysis was performed to construct a correlation heatmap between the marker gene and 9 immunosuppressive checkpoint molecules, including ITPRIPL1, SIGLEC15, TIGIT, CD274 (PD-L1), HAVCR2 (TIM-3), PDCD1 (PD-1), CTLA4, LAG3, and PDCD1LG2 (PD-L2).

### 2.7 Construction of pathway-relation-network

Correlations between key genes and other genes were obtained through biological general repository for interaction datasets (BIOGRID), and were noted as biomarker-target relationship. Key gene-pathway relationships were obtained through KEGG database. The Key gene-pathway network was constructed by integrating the above two relationship, and was visualized via Cytoscape software (v 3.7.2) (Shannon et al., 2003). The study explored key gene interactions via GeneMANIA (http://www.genemania.org/), such as physical interactions, co-expression and pathway (Baiocchi et al., 2016).

### 2.8 Molecular regulatory network analysis

Firstly, based on TCGA-LUAD, DESeq2 package (v 3.4.1) was utilized to uncover the differentially expressed microRNAs (DE-miRNAs) and differentially expressed long noncoding RNAs (DE-lncRNAs) between LUAD and control samples (|log2FC|>0.5, adj. p-value<0.05). After that, the prediction of miRNAs that specifically target the key genes were undertaken through the miRWalk database (http://mirwalk.uni-hd.de/), and the intersection of the DE-miRNAs and the predicted miRNAs was then taken as the target miRNAs for the key genes. Utilizing the miRNet database (https://www.mirnet.ca), the lncRNAs that exhibited associations with the targeted miRNAs were identified, and the intersection of the DE-lncRNAs and the predicted lncRNAs was taken as the target lncRNAs. Subsequently, miRNAs were screened for an opposite expression trend to that of mRNAs, and lncRNAs screened for an opposite expression trend to that of miRNAs. Ultimately, the regulatory network namely, ‘mRNA-miRNA-lncRNA’ was established utilizing ggalluvial package (v 0.12.5) (https://www.mirnet.ca). To explore the transcription factors (TFs) that regulate key genes, TFs were obtained from the NetworkAnalys database (http://www.networkanalyst.ca), after which the regulatory network of TFs-mRNA was visualized by Cytoscape (v 3.7.2).

### 2.9 Molecular docking and molecular dynamics (MD) simulations1

In this study, the two-dimensional and three-dimensional structure of OA and active ingredient structures was accessed from the Pubchem database (https://pubchem.ncbi.nlm.nih.gov/). Additionally, small molecules were examined for charge-balanced, rotatable bonds utilizing AutoDock Tools (v 1.5.7) (Morris et al., 2009). Molecular docking between OA and the key target proteins was then performed, and the free binding energy was evaluated via AutoDock vina (v 1.2.2) (Trott and Olson, 2010). For TOP2A-OA docking, the crystal structure of TOP2A (PDB ID: 1ZXM) was retrieved from the Protein Data Bank. Protein preparation was performed using AutoDock Tools (v 1.5.7), including removal of all crystallographic water molecules, protonation and addition of hydrogen atoms to amino acid side chains at physiological pH, and assignment of Kollman charges, yielding the receptor file 1zxm.pdbqt. The docking grid box was constructed using AutoGrid4 with center coordinates of X = 49.6, Y = 3.766, Z = 22.213, grid dimensions of 126 × 126 × 126 points, and a grid spacing of 0.7083 Å, resulting in a box edge length of approximately 88.54 Å. A distance-dependent dielectric constant of −0.1465 was applied. For ALOX5AP-OA docking, the crystal structure of ALOX5AP (PDB ID: 6VGC) was used, with grid center coordinates of X = 10.306, Y = −0.031, Z = 29.81, grid dimensions of 126 × 100 × 126 points, and a spacing of 0.8472 Å. Ten independent docking runs were performed using AutoDock 4.2 with the Lamarckian Genetic Algorithm, employing a population size of 150, a maximum of 2,500,000 energy evaluations, and 27,000 generations, combined with Solis and Wets local search. Conformational clustering was performed with an RMSD threshold of 2.0 Å, and the conformation with the lowest binding free energy and the largest cluster size was selected as the optimal binding pose. The molecular interactions were visualized via the PyMOL software (v 2.5) (Seeliger and de Groot, 2010). To rigorously benchmark the docking results and avoid arbitrary absolute thresholds (e.g., −5 kcal/mol), well-established known ligands were incorporated as positive controls. Specifically, Doxorubicin and Etoposide were utilized as reference inhibitors for TOP2A, while Veliflapon (AM103) served as the reference for ALOX5AP. It should be emphasized that these docking simulations serve strictly as theoretical supportive evidence for potential binding rather than confirmatory proof of biological interaction.

To further evaluate the dynamic stability of the predicted protein-ligand complexes, MD simulations were performed using GROMACS 2024.4 with the AMBER99SB-ILDN force field and TIP3P water model. The simulation box was set as a cube with a 1 nm distance between the protein surface and the box edge, and counterions were added to neutralize the system. Energy minimization was performed using the steepest descent method, followed by NVT and NPT equilibration using the V-rescale thermostat at 300 K with a 2 fs time step, each for 100 ps? A 100 ns production run was subsequently conducted. Key metrics including Root Mean Square Deviation (RMSD), Root Mean Square Fluctuation (RMSF), total energy, and hydrogen bond distances were analyzed.

### 2.10 Animal experiment

All animal procedures were reported in accordance with the ARRIVE guidelines (Kilkenny et al., 2010). Female BALB/c-nu mice (6–8 weeks old) were purchased from Beijing Weitonglihua Laboratory Animal Technology Co., Ltd. and raised in an SPF-level animal laboratory. The environmental parameters of the laboratory are as follows: temperature 20 °C–26  C, relative humidity 40%–70%, maintaining a 12-h/12-h bright and dark circulation, and the ventilation system meets the SPF standard. Nude mice can freely consume sterilized pellet feed and sterile drinking water. All animal protocols were approved by the Animal Ethics Committee of Yunnan Bestai Biotechnology Co., Ltd. (No. BST-PZ-MICE-20240325-01) and performed following the 3R principles.

Oleanolic acid (OA, purity ≥98% as determined by HPLC) was purchased from Solarbio Life Sciences Co., Ltd., Beijing, China. After 1 week of acclimatization, 24 nude mice were randomly divided into four groups via computer-generated random numbers, with six mice in each group. The sample size was determined based on published literature using comparable experimental protocols. Olive oil in the control group, low dose respectively oleanolic acid group (Low-OA, 37.5 mg/kg, equivalent to 82.1 μmol/kg) group, moderate doses of oleanolic acid (Medium-OA, 75 mg/kg, equivalent to 164.2 μmol/kg) group and high dose of oleanolic acid (High-OA, 150 mg/kg, equivalent to 328.4 μmol/kg). It should be noted that the administered doses, particularly the high dose of 150 mg/kg, were specifically established as mechanistic probes. The primary objective of utilizing this dosage range was to ensure sufficient tissue exposure for the robust evaluation of downstream molecular targets and regulatory pathways, rather than serving as a direct clinical dosing recommendation. Based on the current research foundation, we selected A549 cells to establish a mouse xenograft tumor model. The cell suspension (1 × 10^7^ cells/mL) was inoculated subcutaneously into the right axilla of nude mice, with 0.1 mL injected into each mouse. After inoculation, the general condition of the nude mice was observed daily, including aspects such as activity, hair, body shape and spirit. Following tumor formation, varying concentrations of oleanolic acid or olive oil were administered via gavage, in accordance with the respective experimental groupings. The oleanolic acid solution was freshly prepared before use to ensure its activity and stability. The body weight and tumor size of nude mice were recorded every 2 days. To minimize observer bias, both administration and subsequent endpoint evaluations, including tumor volume and weight measurements, were conducted by independent investigators strictly blinded to the group assignments. After the experiment, samples were taken to measure the mass and volume of the tumor. The tumor volume is calculated by the following formula: Tumor volume (mm^3^) = (length) × (width)^2^ × 0.5.

### 2.11 Western blot analysis

Western blot analysis was performed to detect protein expression in mouse tumor tissues. Tumor tissues were homogenized in a tissue grinder and incubated with RIPA lysis buffer containing protease and phosphatase inhibitors on ice for 10 min. The lysate was centrifuged at 12,000 rpm for 10 min at 4 °C, and the supernatant was collected for protein quantification using a BCA Protein Assay Kit (Protein, United States). After denaturation, proteins were separated by SDS-PAGE and transferred to a PVDF membrane, which was then blocked with a solution containing 5% skim milk. The membrane was incubated overnight at 4 °C with primary antibodies against β-actin (1:1,000, Affinity, United States), TOP2A (1:2000, Protein, United States), and ALOX5AP (1:3,000, Abcam, United States). After washing with TBST, the membrane was incubated with secondary antibodies at room temperature for 2 h. Antibody reactions were detected using ECL substrate (Yeasen, Shanghai, China), and protein bands were analyzed with ImageJ software.

### 2.12 Preliminary assessment in a small clinical cohort

To explore the clinical expression of the screened biomarkers, qPCR assessment was conducted on samples from 20 patients with LUAD. These samples were all obtained from July 2023 to July 2024. All of these samples were obtained through surgical procedures involving precise dissection. According to the standard that the distance from the cancer tissue was greater than 3 cm, the LUAD specimens were divided into cancer tissues and adjacent tissues. All the enrolled patients were diagnosed with LUAD by histopathology and had not received any treatment before participating in this study. This study followed the principles of the Helsinki Declaration and was approved by the Clinical Research Ethics Committee of the First Affiliated Hospital of Kunming Medical University (kmmu2021172). All subjects signed the informed consent form before participating in the study. Data analysis was conducted using the 2^(-ΔΔCt) method, with three separate qPCR experiments carried out. The primer sequences employed for the internal reference gene β-actin in the experiment are as follows: forward primer-CCATCACCATCTTCCAGGAGCGA, and reverse primer-GGATGACCTTGCCCACAGCCTTG. For the TOP2A gene, the primer sequences are: forward primer-GCCAGAATCTGTTCGGTTCAAC, and reverse primer-AGGAAACTGAGTGCCGGCTT. The primer sequences for the ALOX5AP gene are: forward primer-CTGCGTTTGCTGGACTGATG, and reverse primer - GGA​GAT​GGT​GGT​GGA​GAT​CG.

### 2.13 Statistical analysis

Data are presented as Mean ± SEM. GraphPad Prism 9.5 and R software (v 4.3.1) were utilized for statistical analysis. The Wilcoxon test was employed to compare the two groups. p < 0.05 was considered statistically significant. No artificial intelligence tools were utilized in any stage of this research.

## 3 Results

### 3.1 Screening and enrichment analysis of candidate genes

The technical route is shown in Figure 1. The two-dimensional (2D) and three-dimensional (3D) structures of OA are shown in Supplementary Figure S1A. Enrichment analyses (Supplementary Figures S1B,C) indicated that 100 predicted OA-target genes are significantly enriched in four functional clusters of pathways: transcriptional regulation and signal transduction (e.g., Nuclear Receptor transcription pathway), metabolic regulation (e.g., Metabolism of lipids), inflammation and immune response (e.g., regulation of inflammatory response), and environmental and hormone response (e.g., response to hormone). Notably, the Nuclear Receptor transcription pathway and lipid metabolism are the most prominent, reflecting OA’s multi-dimensional functional involvement via its various target genes.

**FIGURE 1:**
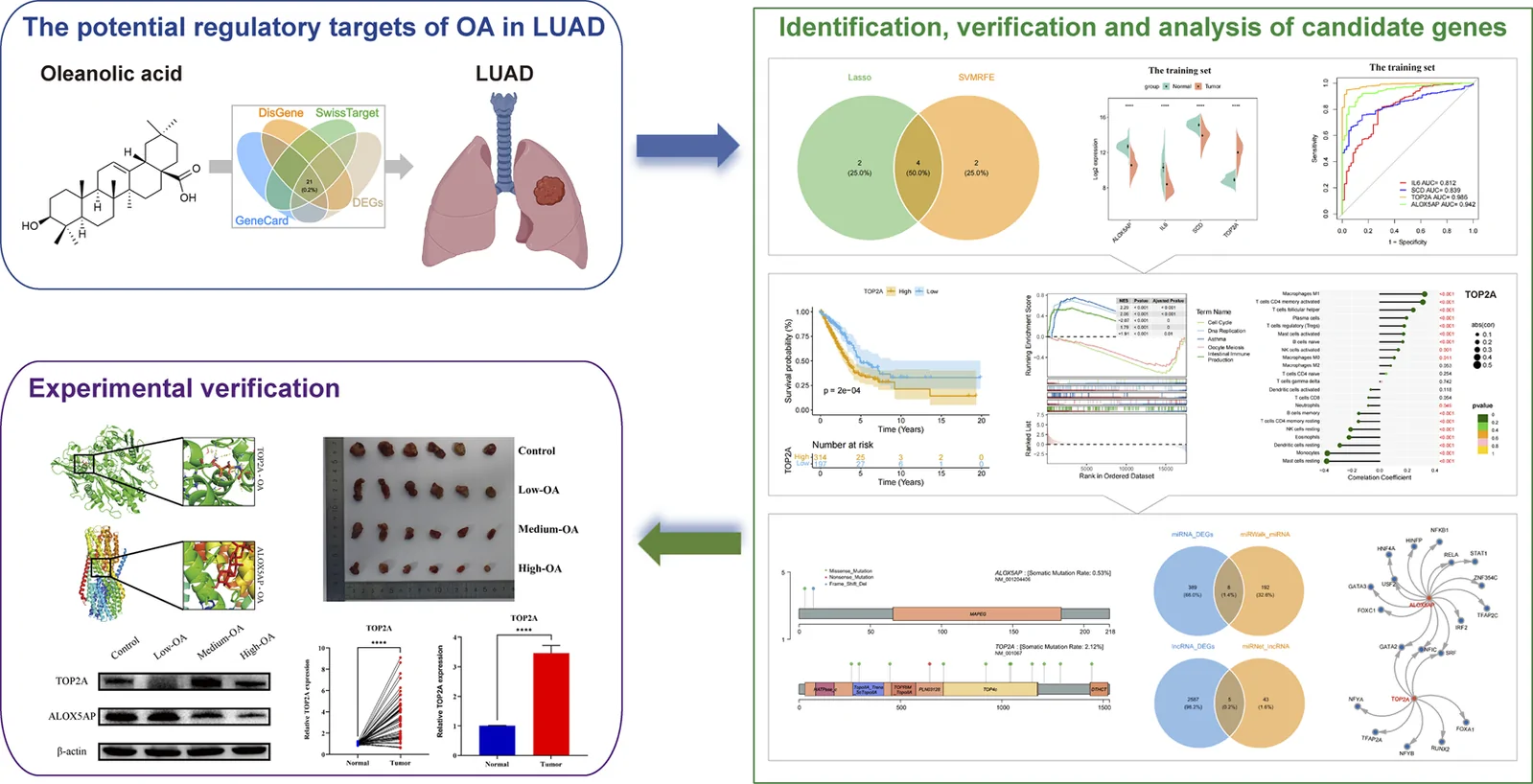
Flowchart of OA’s anti-tumor effect in LUAD: Based on network pharmacology and machine learning.

In TCGA-LUAD cohort, a total of 9,508 DEGs were identified between tumor and normal groups, with 6,069 genes significantly upregulated and 3,439 genes notably downregulated. The volcano plot (Figure 2A, left) vividly displays these DEGs, highlighting the top five upregulated (e.g., PYCR1, FAM83A) and downregulated (e.g., OTUD1, EPAS1) genes by their statistical significance (−log10 (Adj.PValue)) and expression fold change (log2FoldChange). Meanwhile, the heatmap (Figure 2A, right) illustrates the expression distribution of DEGs between tumor and normal samples, further supporting the distinct transcriptional patterns in LUAD. Additionally, by intersecting OA target genes, LUAD-related genes, and DEGs, the study obtained 21 candidate genes associated with OA and LUAD (Figure 2B).

**FIGURE 2:**
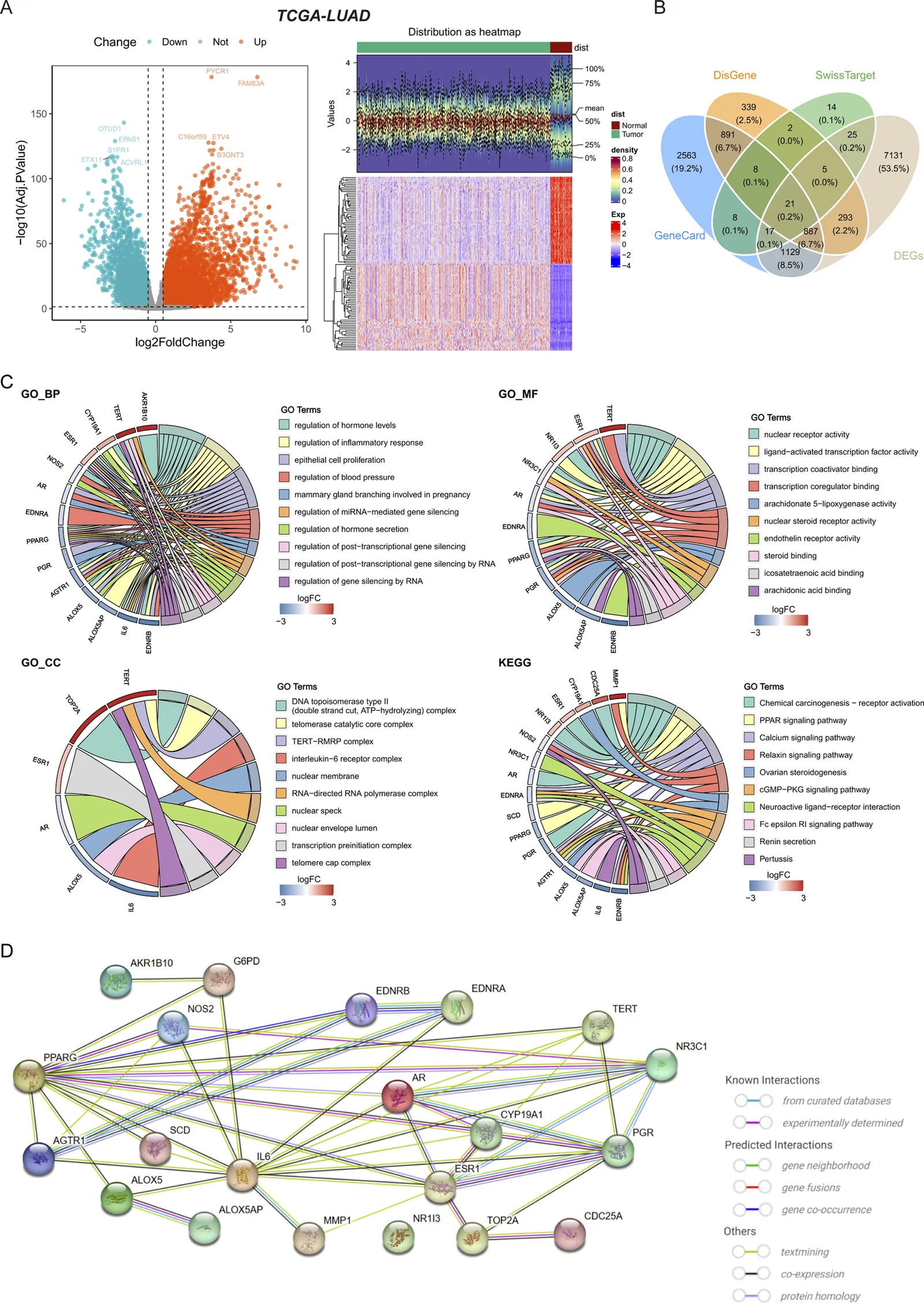
Identification and functional enrichment of candidate genes. **(A)** Volcanic maps and heatmap of DEGs between lung adenocarcinoma and normal samples in the TCGA-LUAD dataset. “Down” indicates a down-regulated gene, “Up” indicates an up-regulated gene, and “Not” indicates no differential expression of the gene. **(B)** Venn diagram of candidate genes. **(C)** GO and KEGG enrichment analysis of candidate genes. The left side of each figure corresponds to the gene and its up-down-regulation relationship, while the right side corresponds to the important pathway or functional name. **(D)** PPI network of the 21 candidate genes.

To illustrate the functional landscape of the 21 candidate genes, enrichment analysis and PPI network analysis were performed. In terms of enrichment (Figure 2C), these genes were significantly involved in multiple GO categories: biological processes (GO_BP) such as regulation of hormone levels, inflammatory response regulation, and cell proliferation regulation; molecular functions (GO_MF) including nuclear receptor activity and ligand-activated transcription factor activity; and cellular components (GO_CC) like DNA topoisomerase complex and nuclear membrane. Key KEGG pathways, such as PPAR signaling and neuroactive ligand-receptor interaction, were also enriched. Meanwhile, the PPI network (Figure 2D) revealed a complex interaction pattern among these genes, with hub genes like PPARγ, IL6, and AR mediating diverse interactions (e.g., known database interactions, experimental evaluations) and participating in coordinated regulation of metabolism, inflammation, and other biological processes.

### 3.2 Identification and evaluation of biomarkers

A total of six feature genes were screened by LASSO regression analysis, including IL6, EDNRB, SCD, MMP1, TOP2A, and ALOX5AP (lambda.min = 0.0028) (Figure 3A). In SVM-RFE analysis, six feature genes were obtained (error rate of 0.00894, accuracy of 0.991), including ALOX5AP, TOP2A, SCD, IL6, ALOX5 and G6PD (Figure 3B). Then, the intersection of these two analysis algorithms was taken to obtain four candidate key genes, which were ALOX5AP, TOP2A, SCD and IL6 (Figure 3C). Furthermore, Wilcoxon test identified that ALOX5AP, SCD and IL6 were downregulated genes, while TOP2A was upregulated gene across both the training and testing cohorts. ALOX5AP and TOP2A exhibited significantly disparities between LUAD and control groups, and the trends remained consistent within the two sets (Figure 3D). The ROC curve revealed that the AUC values of ALOX5AP and TOP2A were exceeded 0.9 in both sets, indicating excellent diagnostic results. Synthesizing the results, ALOX5AP and TOP2A were defined as biomarkers in this study (Figure 3E). Paired-sample analysis in the TCGA-LUAD dataset (Figure 3F) also revealed significant differential expression of ALOX5AP and TOP2A between tumor and matched adjacent normal tissues. We further explored their protein–level expression via the CPTAC database (Figure 3G), consistently revealing that ALOX5AP was significantly downregulated while TOP2A was markedly upregulated in tumor samples. Specifically, ALOX5AP was markedly downregulated (p < 2.2e-16) in tumor tissues, while TOP2A was significantly upregulated (p < 2.2e-16) in tumor tissues compared to their paired normal counterparts, further supporting their distinct expression patterns in LUAD.

**FIGURE 3:**
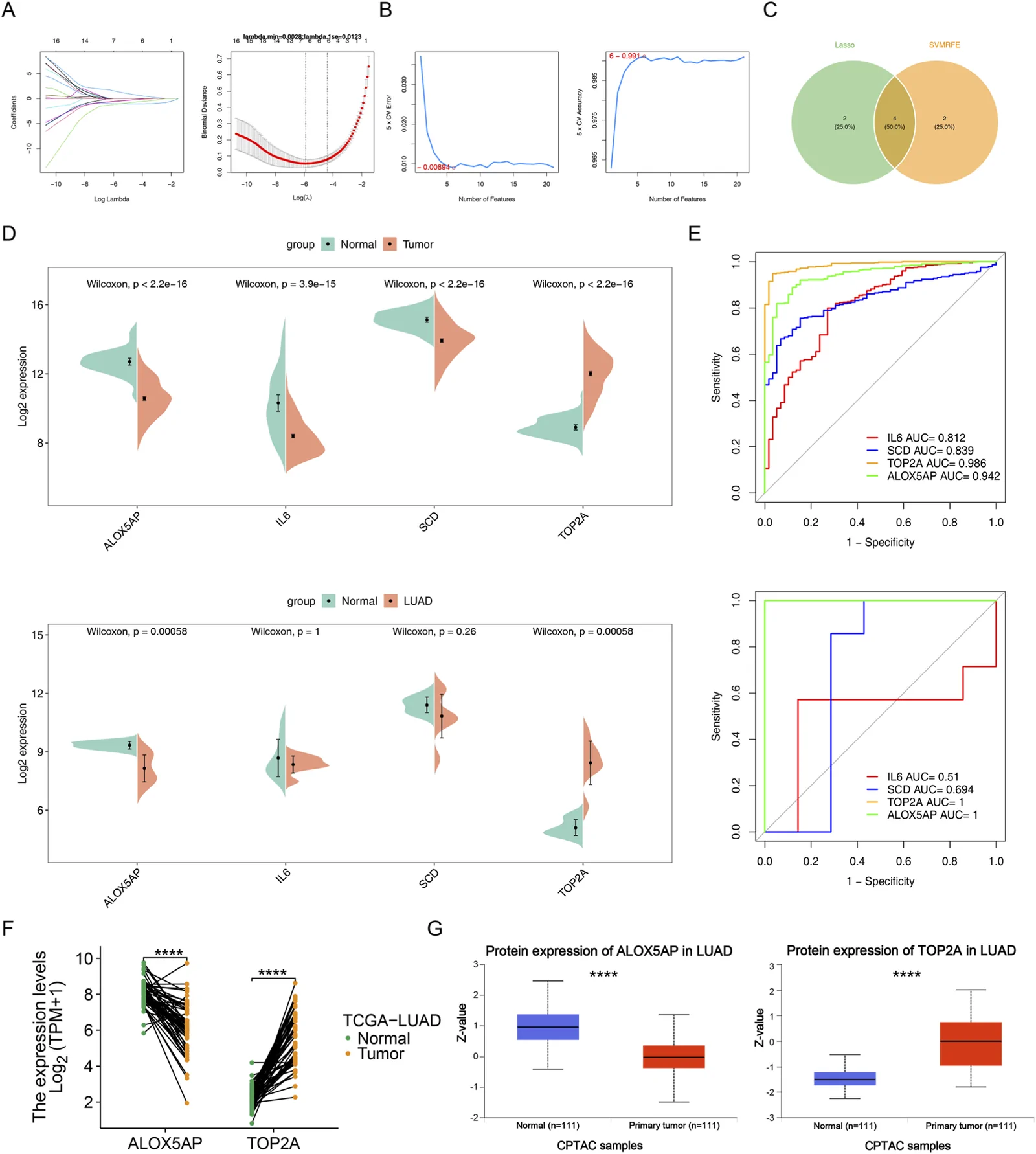
Identification of biomarkers of OA in LUAD. **(A)** Lasso-cox regression analysis graph. **(B)** Generalization error and Feature quantity graph of SVM-RFE algorithm. **(C)** Venn diagram of candidate biomarkers. **(D)** Expression validation of candidate biomarkers in training sets and validation sets. **(E)** ROC curves of candidate biomarkers. **(F)** Gene expression levels of the 2 prognostic biomarkers in matched TCGA-LUAD samples. **(G)** Protein expression levels of the 2 prognostic biomarkers in the CPTAC dataset (via UALCAN database). ns, not statistically different, *p < 0.05, **p < 0.01, ***p < 0.001, ****p < 0.0001.

### 3.3 Prognostic analysis of ALOX5AP and TOP2A

Using optimal cut-off values for stratification, survival analysis of 511 TCGA-LUAD patients revealed that both low ALOX5AP expression and high TOP2A expression were significantly associated with poor OS (ALOX5AP: exact p = 0.0097, HR = 1.46, 95% CI: 1.094-1.949; TOP2A: exact p = 0.0002, HR = 0.553, 95% CI: 0.403–0.758; Figure 4A). Clinicopathological feature analysis revealed (Figure 4B) that the expression level of ALOX5AP was higher in the ≥65 years age group, female group, and alive group. In contrast, TOP2A expression was higher in the <65 years age group, male group, and dead group.

**FIGURE 4:**
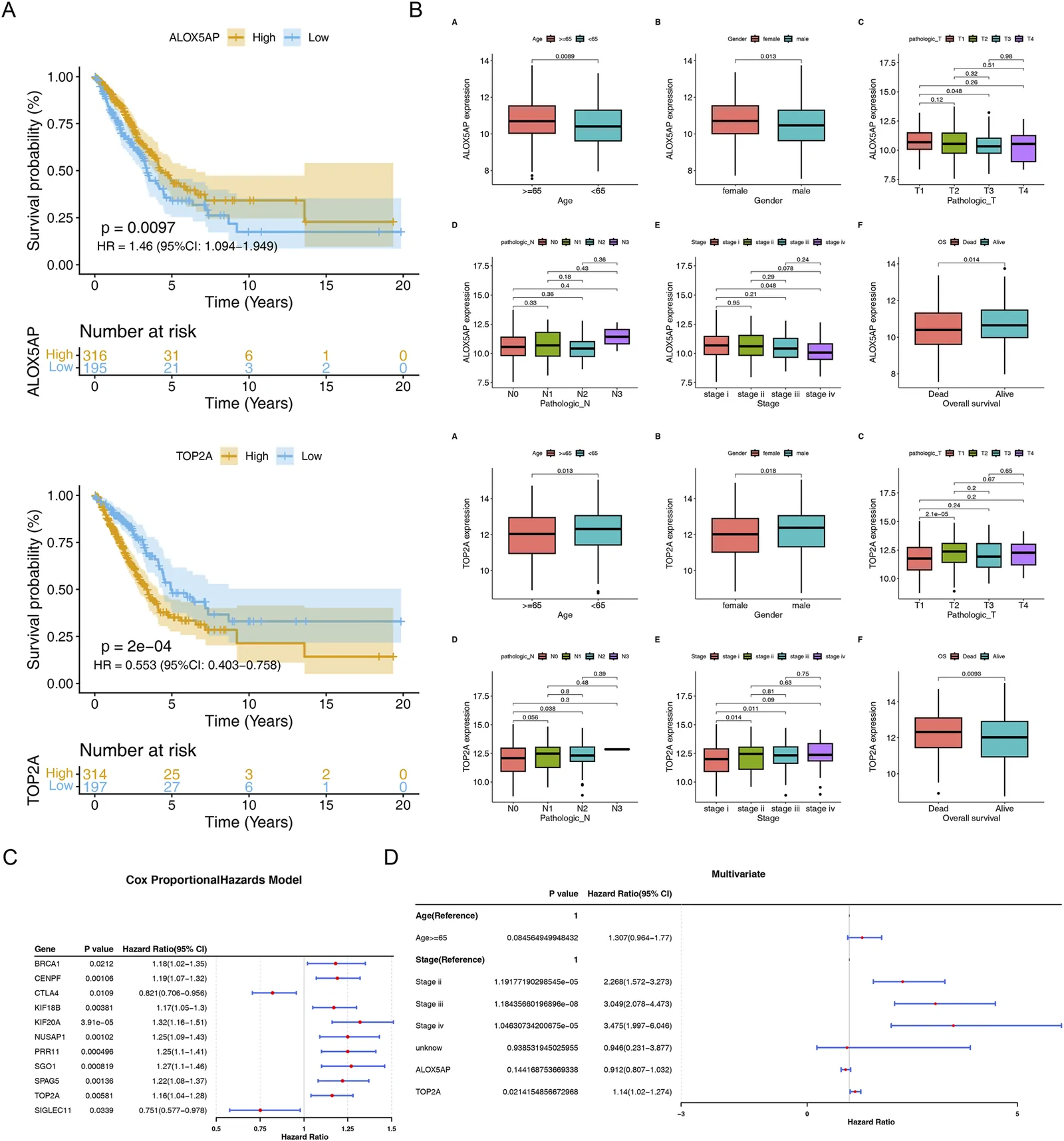
Prognostic value and clinicopathological feature analysis of the identified biomarkers in LUAD. **(A)** Kaplan-Meier survival curves of TOP2A and ALOX5AP in the TCGA-LUAD dataset. “High” represents the group with high gene expression, and “Low” represents the group with low gene expression. **(B)** Association analysis of clinicopathological features of TOP2A and ALOX5AP. pathologic_T, pathological tumor size; pathologic_N, pathological lymph node metastasis; stage, TNM stage (Tumor-Node-Metastasis stage); OS, Overall Survival. **(C)** Forest plot detailing the univariate Cox proportional hazards regression analysis of selected candidate genes, illustrating their individual Hazard Ratios (HR) and 95% Confidence Intervals (CI) for OS. **(D)** Forest plot representing the refined multivariate Cox proportional hazards regression analysis incorporating age, clinical stage, ALOX5AP, and TOP2A. The analysis identifies clinical stage and TOP2A expression as independent prognostic risk factors for OS, whereas ALOX5AP does not maintain independent prognostic significance after adjusting for these covariates. ns, not statistically different, *p < 0.05, **p < 0.01, ***p < 0.001, ****p < 0.0001.

When stratified by the median expression level of LUAD, TOP2A remained significantly associated with prognosis (p = 0.0068), whereas ALOX5AP showed no statistically significant correlation with OS (p = 0.078, Supplementary Figure S2A). Consistently, in GEPIA2 database analysis of LUAD tumor samples using median-based stratification, TOP2A retained its significant prognostic value (p = 0.011), while ALOX5AP failed to show a meaningful association with survival outcomes (Supplementary Figure S2B). In contrast, median-based survival analysis in the KM Plotter database revealed that both genes were significantly correlated with prognosis in LUAD, with p-values all less than 0.0001 (Supplementary Figure S2C).

To further rigorously evaluate the independent prognostic value and adjust for potential confounders, a Cox proportional hazards regression model was constructed (Figure 4C). The Cox regression analysis demonstrated that TOP2A remains a significant and independent risk factor for OS in LUAD (p = 0.00581, Hazard Ratio [HR] = 1.16, 95% CI: 1.04–1.28). In contrast, ALOX5AP did not exhibit sustained independent prognostic significance in the context of comprehensive multivariate adjustment, highlighting the distinct prognostic robustness between the two biomarkers. To rigorously evaluate the independent prognostic value of these biomarkers and adjust for potential clinical confounders, a multivariate Cox proportional hazards regression model was constructed incorporating age, clinical stage, ALOX5AP, and TOP2A expression (Figure 4D). The multivariate analysis demonstrated that TOP2A remains a significant and independent prognostic risk factor for OS in LUAD (p = 0.0214, HR = 1.14, 95% CI: 1.02–1.274), independent of tumor stage and age. In stark contrast, ALOX5AP did not maintain independent prognostic significance after adjusting for these clinical covariates (p = 0.144, HR = 0.912, 95% CI: 0.807–1.032), highlighting a distinct divergence in prognostic robustness between the two targets.

### 3.4 GSEA enrichment analysis of ALOX5AP and TOP2A in LUAD

The GSEA enrichment analysis results revealed distinct functional profiles for ALOX5AP and TOP2A. Specifically, ALOX5AP was predominantly enriched in HALLMARK pathways including Allograft Rejection, Interferon Gamma Response, and Inflammatory Response, with significant enrichment in KEGG pathways such as Leishmania Infection and Hematopoietic Cell Lineage (Figure 5A). These findings suggest that ALOX5AP is primarily involved in immune-inflammatory processes, including transplant rejection, interferon-mediated immune responses, and cytokine pathway regulation, and may also participate in epithelial-mesenchymal transition.

**FIGURE 5:**
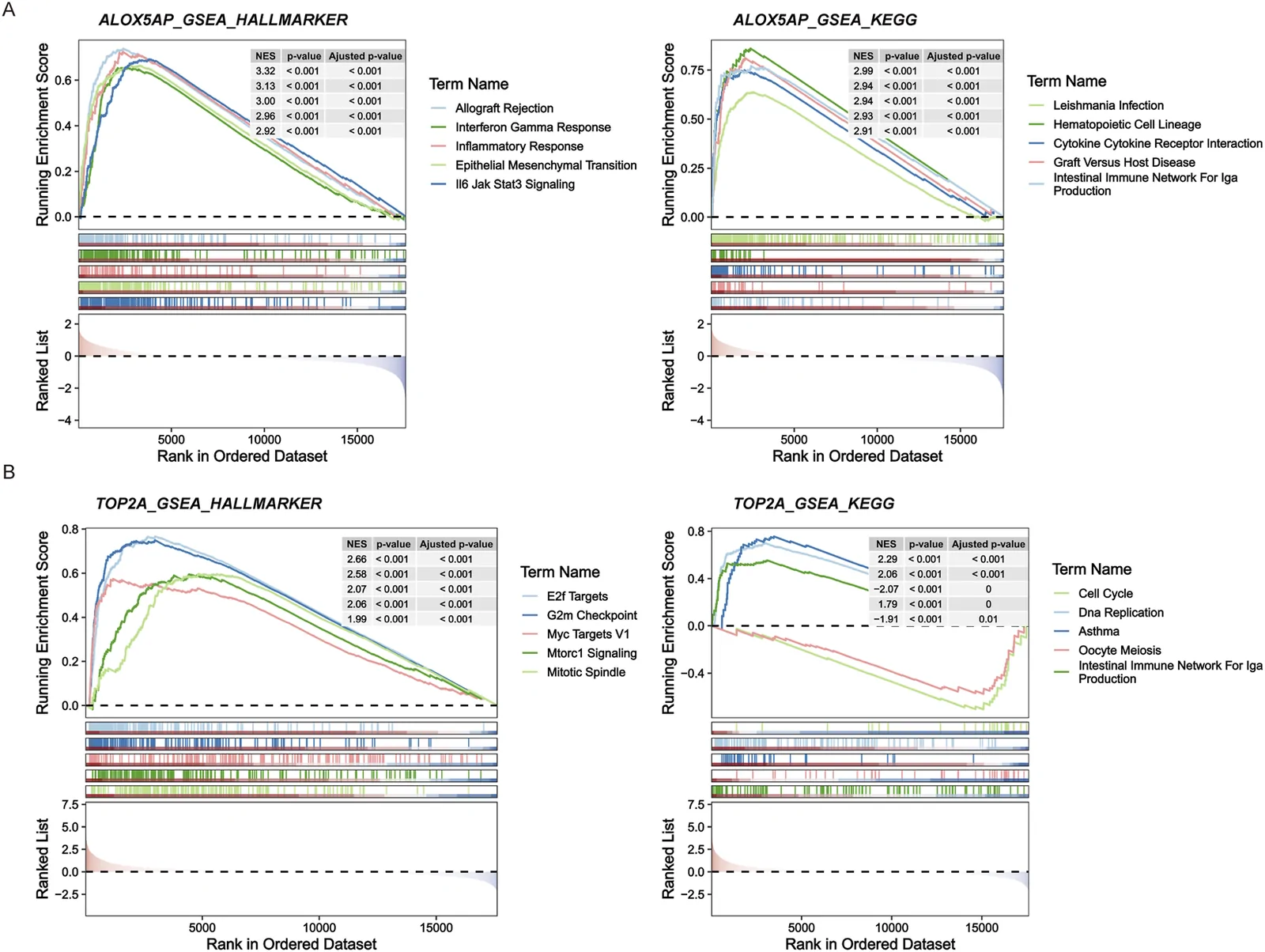
Gene-set enrichment analysis (GSEA) of TOP2A and ALOX5AP in LUAD. **(A)** GSEA plots for ALOX5AP using the HALLMARK (left) and KEGG (right) gene sets. **(B)** GSEA plots for TOP2A using the HALLMARK (left) and KEGG (right) gene sets. For each analysis the top five enriched pathways are listed; NES, normalized enrichment score; FDR, false-discovery rate.

In contrast, TOP2A showed enrichment in HALLMARK pathways such as E2F Targets and G2M Checkpoint, and was notably enriched in KEGG pathways including Cell Cycle and DNA Replication (Figure 5B), indicating that its core function is closely linked to cell cycle regulation and proliferation, serving as a key molecule in cell division and tumor proliferation. Notably, both ALOX5AP and TOP2A were co-enriched in the “Intestinal Immune Network for Iga Production” and asthma-related pathways (Leukotriene infection, Asthma), implying a potential synergistic role of these two biomarkers in the inflammatory mechanisms underlying intestinal mucosal immune disorders and asthma.

### 3.5 Mutation analysis and immune infiltration analysis of ALOX5AP and TOP2A in LUAD

To ascertain whether the dysregulation of TOP2A and ALOX5AP in LUAD is driven by irreversible genomic alterations or by dynamic transcriptional changes, an analysis of their SNV, CNV, and methylation statuses was initially conducted. Following this, the results of SNV analysis showed (Figure 6A) that the somatic mutation rate of ALOX5AP in LUAD was 0.53%, with mutation types including missense mutations and frame-shift deletions, and its mutation sites were located in the outer region of the MAPEG domain. The somatic mutation rate of TOP2A was 2.12%, with mutation types including missense mutations and nonsense mutations, and the mutation sites were distributed in multiple domains such as HATPase_c, TopoIIA_Trans_ScTopoIIA, and TOPRIM_TopoIIA, which are closely related to DNA topoisomerase activity and cell cycle regulation. Further survival analysis demonstrated that the SNV mutation status of TOP2A was significantly associated with shortened OS, disease-specific survival (DSS), and progression-free survival (PFS) in patients (p < 0.05), but showed no statistical correlation with disease-free survival (DFI) (Figure 6B, Supplementary Figure S3A). Additionally, neither copy number variation (CNV) nor methylation status of ALOX5AP or TOP2A exhibited significant correlations with the aforementioned four prognostic outcomes (Supplementary Figures S3B,C).

**FIGURE 6:**
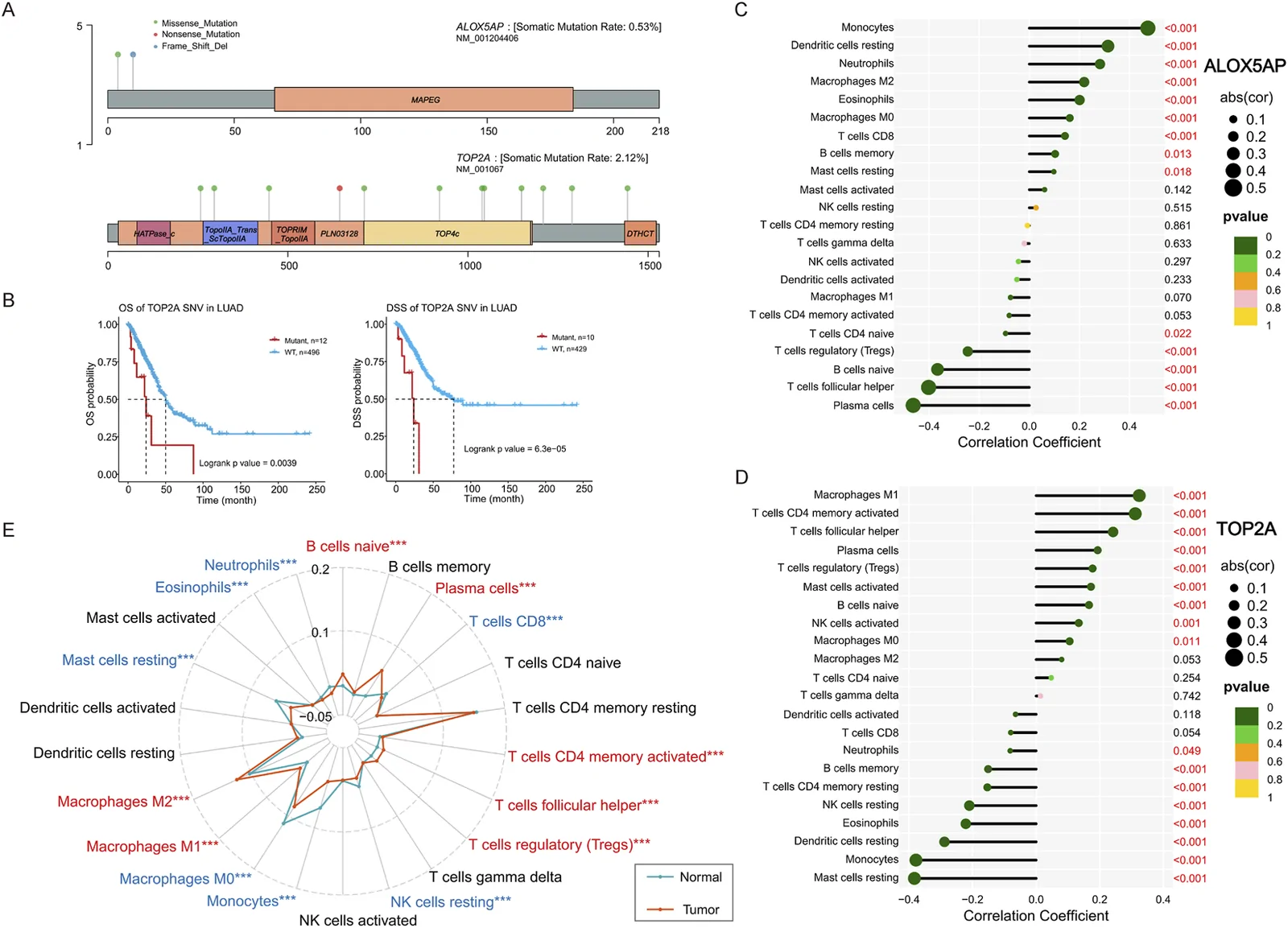
Mutation landscape and immune infiltration analysis of TOP2A and ALOX5AP in LUAD. **(A)** Somatic alteration frequencies of TOP2A and ALOX5AP in LUAD (GSCA database, n = 513). **(B)** Kaplan-Meier survival curves of TOP2A SNV status in LUAD patients (OS, left; DSS, right). **(C,D)** The correlation between TOP2A and ALOX5AP and immune cells. **(E)** Radar plot comparing mean infiltration of 22 immune-cell subpopulations between TCGA-LUAD tumor (red) and adjacent normal (blue) tissues. *p < 0.05, **p < 0.01, ***p < 0.001, ****p < 0.0001.

In LUAD, ALOX5AP expression is significantly positively correlated with Dendritic cells resting, Neutrophils, and Macrophages M2, and negatively correlated with Plasma cells and T cells follicular helper (p < 0.001, Figure 6C). In contrast, TOP2A expression is positively associated with Macrophages M1, T cells CD4 memory activated, and T cells follicular helper, and negatively correlated with resting Mast resting cells and Monocytes (p < 0.001, Figure 6D). This suggests that ALOX5AP may contribute to immune suppression in LUAD, whereas TOP2A is likely involved in immune cell recruitment and regulation. Further analyses in LUAD tumor samples (Supplementary Figures S4A,B) provided additional support for this hypothesis. Consistent with this, ESTIMATE analysis (Supplementary Figure S4C) revealed contrasting associative patterns of ALOX5AP and TOP2A with the immune microenvironment. Regarding immune checkpoints (Suppl_Fig4D), ALOX5AP showed significantly positive correlations with most immunosuppressive checkpoint molecules (R > 0.3, p < 0.001), whereas TOP2A showed distinct association patterns with these checkpoints, with much weaker correlation strengths. Immune infiltration comparison between LUAD tumor and normal tissues (Figure 6E), reveals significant differences in 14 out of 22 immune cell types (p < 0.001). Tumor tissues exhibit higher infiltration of naive B cells, plasma cells, M1/M2 macrophages, and Tregs, suggesting an altered immune profile potentially associated with adaptive immune cell presence and markers of evasion. Normal tissues have higher levels of neutrophils and resting NK cells, which theoretically aligns with innate immune surveillance characteristics.

### 3.6 Pathway network relationship

To explore the comprehensive interaction landscape, an initial key gene-pathway-shared target network was constructed (Supplementary Figure S5). However, to mitigate network inflation and prioritize biologically interpretable modules, the presentation of global, highly dense interaction networks was deliberately omitted. Instead, a highly focused, simplified regulatory network was constructed strictly centered on the identified biomarkers, ALOX5AP and TOP2A. As depicted in the revised schematic diagram (Figure 7A), this focused network highlights the theoretical upstream TF regulatory axes. The simplified network reveals that ALOX5AP and TOP2A share three critical upstream transcription factors: SRF, NFIC, and GATA2 (indicated by the central shared nodes). By narrowing the scope exclusively to these specific regulatory nodes, this focused analysis is intended solely to visualize theoretical macroscopic associations, rather than asserting definitive, comprehensive mechanistic pathways. Further analysis revealed that biomarkers were significantly involved in the biosynthetic and metabolic pathways of icosanoids, as well as the specific metabolic processes of leukotrienes (Figure 7B). Regulatory network analysis provided critical insights into the underlying mechanisms of biomarkers regulation. By intersecting differentially expressed miRNAs (DE-miRNAs) with predicted miRNAs, we identified 8 significant miRNAs (Figure 7C). Similarly, the intersection of differentially expressed lncRNAs (DE-lncRNAs) with predicted lncRNAs yielded 5 key lncRNAs. The regulatory networks highlighted three important interactions: TOP2A-hsa-miR-378c-LINC00174, TOP2A-hsa-miR-378c-LINC00958, and TOP2A-hsa-miR-378c-NEAT1 (Figure 7D).

**FIGURE 7:**
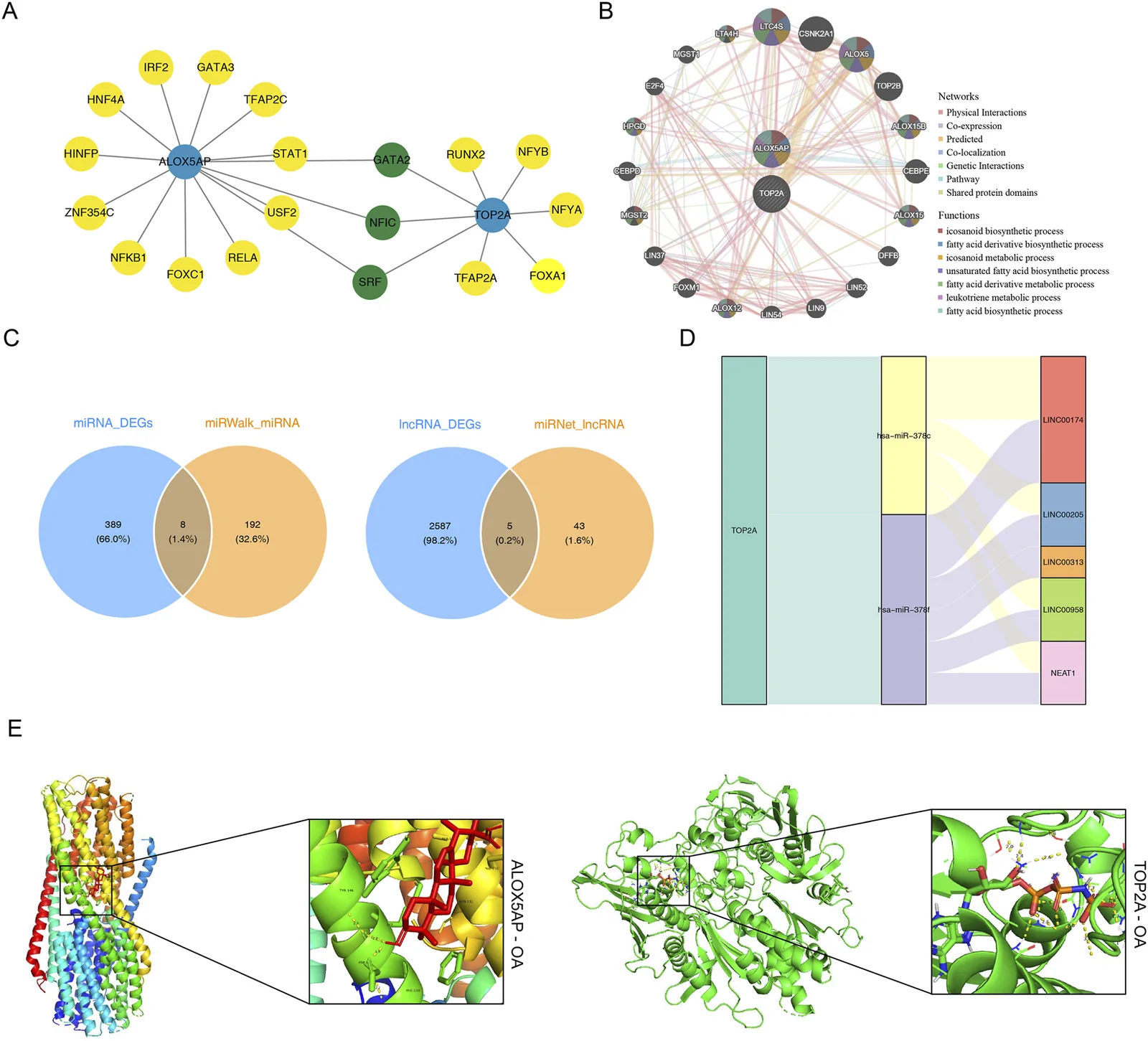
The potential mechanism of OA in LUAD. **(A)** Simplified schematic of the prioritized transcription factor (TF) regulatory network. **(B)** GeneMANIA analysis of TOP2A and ALOX5AP. **(C)** Venn diagram of intersections between differentially expressed and predicted miRNAs/lncRNAs. **(D)** Molecular regulatory network of TOP2A. **(E)** Molecular docking between OA and TOP2A/ALOX5AP.

Co-expression relationships among ALOX5AP, TOP2A, and the three transcription factors were further investigated (Supplementary Figure S6A). ALOX5AP exhibited a weak positive correlation with NFIC (R = 0.260, p < 0.001), while no statistically significant association was observed between ALOX5AP and SRF or GATA2 (R = 0.041, R = 0.039), indicating no correlation between them. In contrast, TOP2A showed a weak positive correlation with SRF (R = 0.243, p < 0.001) and a weak negative correlation with NFIC (R = −0.142, p < 0.01). No statistically significant association was found between TOP2A and GATA2 (R = −0.017), suggesting no correlation between the two. No significant correlation was detected between ALOX5AP and TOP2A (R = −0.065, p = 0.141, Supplementary Figure S6B). Differential expression analysis revealed that all three transcription factors were specifically downregulated in tumor tissues relative to normal counterparts in both unpaired and paired TCGA-LUAD samples (p < 0.001, Supplementary Figure S6C). However, their individual expression levels were not significantly associated with OS in LUAD patients (Supplementary Figure S6D).

### 3.7 Molecular docking and MD simulations of the TOP2A and ALOX5AP genes

Molecular docking analysis was employed to elucidate the binding interactions between OA and its target genes. The ALOX5AP-OA interaction exhibited a free binding energy of −7.78 kcal/mol, while the TOP2A-OA interaction showed an even stronger binding affinity with a free energy of −8.96 kcal/mol. These results provide theoretical predictive support for potential interactions between OA and both key genes, suggesting its possible modulatory role (Figure 7E). Detailed analysis of the TOP2A-OA interaction revealed that OA is embedded within a helical-sheet mixed domain of TOP2A, with key interacting residues including LYS, ARG, ASP, and GLU, which form a hydrogen-bond network with distances ranging from 1.6 to 2.5 Å. Additional hydrophobic contacts involving LEU, VAL, ILE, and PHE residues contribute to binding stabilization through van der Waals forces and hydrophobic packing. For the ALOX5AP-OA interaction, the binding is primarily mediated by TYR, ASN, and PHE residues through cooperative hydrogen-bonding and hydrophobic interactions. Notably, the binding pocket compositions differ substantially between the two targets, suggesting that OA employs adaptive binding modes rather than sharing identical binding residues across different proteins. To contextually evaluate the binding affinities, the results were compared against established clinical inhibitors serving as positive controls (Supplementary Figure S7). For ALOX5AP, the reference ligand Veliflapon exhibited a binding energy of −10.3 kcal/mol, while the ALOX5AP-OA interaction demonstrated a competitive theoretical affinity with a binding energy of −7.78 kcal/mol. Regarding TOP2A, the known inhibitors Doxorubicin and Etoposide yielded binding energies of −10.1 kcal/mol and −9.4 kcal/mol, respectively. In comparison, the TOP2A-OA interaction showed a highly comparable binding affinity with a free energy of −8.96 kcal/mol. While the theoretical affinities of OA are slightly lower than those of highly optimized synthetic clinical drugs—a common characteristic of natural pentacyclic triterpenoids—these comparative results demonstrate that OA possesses a biologically plausible binding capacity for both targets. Comparative analysis of the residue interaction profiles between OA and the reference TOP2A inhibitors (Etoposide and Doxorubicin) revealed that common interacting residue—ARG—was shared between OA and the known inhibitors. This minimal overlap suggests that OA and classical TOP2A inhibitors may engage distinct binding determinants within the same general pocket, rather than competing for an identical set of residues. Nevertheless, these *in silico* binding energies must be interpreted strictly as supportive theoretical predictions, which necessitate future biophysical assays to confirm actual functional inhibition.

The RMSD plots showed minimal fluctuations for both TOP2A-OA and OA-ALOX5AP complexes within the 0–50 ns timeframe, suggesting stable binding (Supplementary Figure S8A). RMSF analysis revealed that TOP2A-OA exhibited relatively small fluctuations throughout the simulation, indicating a more rigid binding mode, whereas ALOX5AP-OA displayed markedly larger fluctuations, suggesting greater conformational flexibility (Supplementary Figure S8B). Total energy remained consistently low (below −100,000 kcal/mol) with minimal fluctuations throughout the simulations (Supplementary Figure S8C). Hydrogen bond counts between the ligand and protein remained stable across the 0–100,000 ps trajectory for both complexes, further supporting the stability of the interactions (Supplementary Figure S8D). These dynamic simulation data complement the static docking results by confirming the binding stability of OA with both targets under near-physiological conditions.

### 3.8 *In vivo* animal experiments and preliminary clinical sample assessment

To empirically test the hypothesis generated by the *in silico* predictions and to investigate the actual pharmacological modulation of these targets within a complex biological system, an *in vivo* xenograft model was established. Initial body weight did not differ between groups. Throughout the study no treatment related adverse events such as rapid weight loss, diarrhea, skin ulceration or behavioral abnormalities were recorded and no animals were excluded. OA markedly reduced both volume and weight of LUAD xenografts (Figures 8A–C), with the strongest suppression in the High OA (150 mg/kg, equivalent to 328.4 μmol/kg) group, but pharmacological effect did not increase with dose. Western blot analysis revealed highly distinct pharmacological dynamic profiles concerning the dose-response relationship. TOP2A protein levels were significantly reduced even at the lowest administered dose (37.5 mg/kg, equivalent to 82.1 μmol/kg), with no substantial further decrease observed at higher doses. This saturation effect at a lower concentration indicates that TOP2A acts as a highly sensitive, primary pharmacological target. Conversely, ALOX5AP protein was downregulated only in the supra-pharmacological high-dose group (150 mg/kg, equivalent to 328.4 μmol/kg), suggesting that its modulation requires a significantly higher threshold and may represent a secondary or non-specific dose-response event (Figure 8D). In contrast, TOP2A protein was significantly reduced at the Low OA dose with no further decrease at higher doses, identifying TOP2A as an early molecular target of OA. Analysis of tumor and matched normal tissues from 20 LUAD patients by RT qPCR revealed significantly higher TOP2A mRNA and lower ALOX5AP mRNA in tumors (Figure 8E), suggesting their distinct expression patterns in LUAD.

**FIGURE 8:**
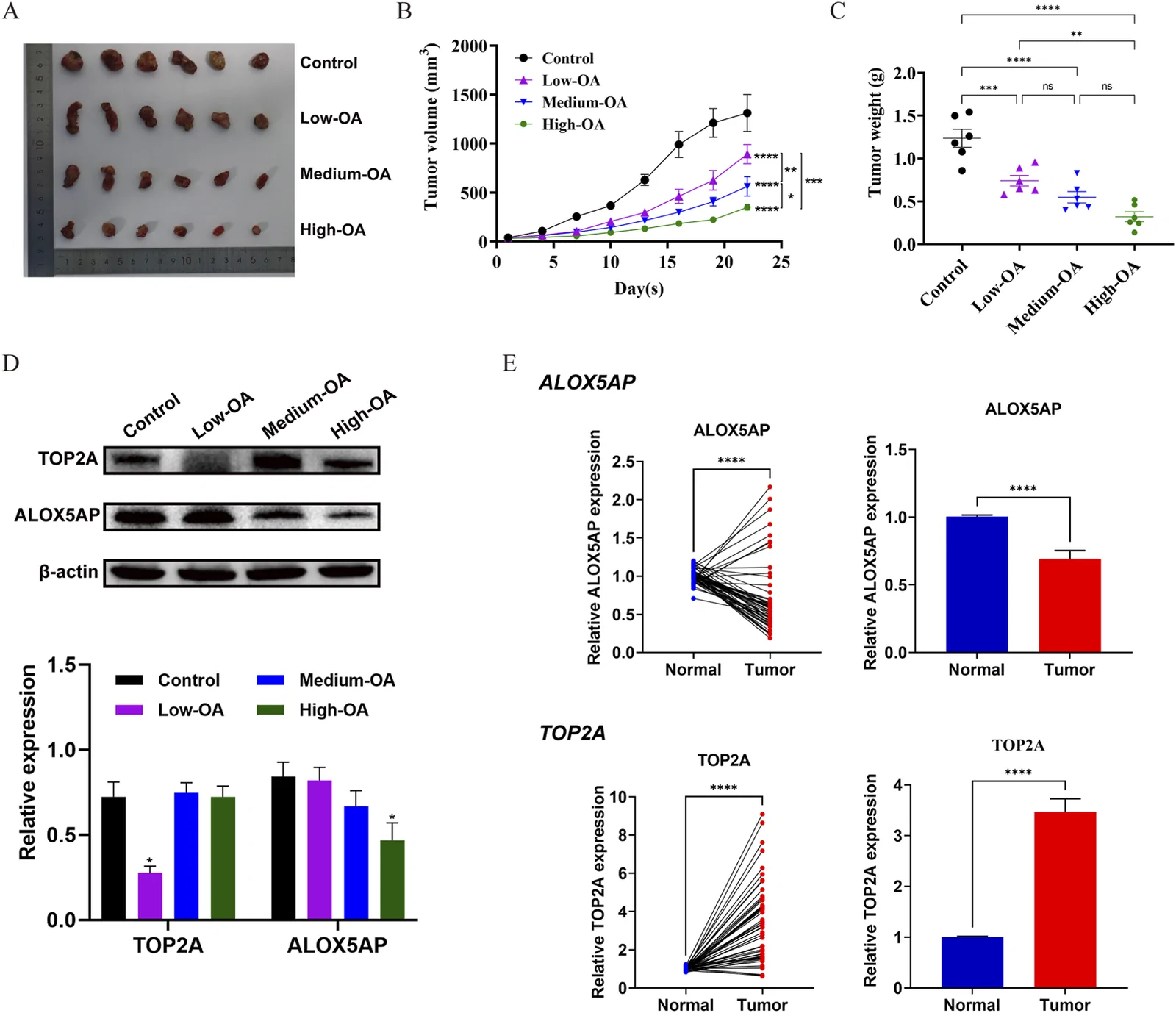
Animal experiments and clinical sample verification. **(A)** Isolated tumor tissues of the Balb/c nude mouse model. **(B)** Detection of tumor volume (mm^3^) of isolated tumors in nude mice. **(C)** Detection of tumor weight (g) of isolated tumors in nude mice. **(D)** Western blotting detection band graph and its quantitative bar graph. **(E)** The mRNA expression of TOP2A and ALOX5AP in 20 patients with LUAD. Low-OA represents the low-dose oleanolic acid group (37.5 mg/kg); Medium-OA represents the medium-dose oleanolic acid group (75 mg/kg); High-OA represents the high-dose oleanolic acid group (150 mg/kg). ns, not statistically different, *p < 0.05, **p < 0.01, ***p < 0.001, ****p < 0.0001.

## 4 Discussion

LUAD exhibits distinct features of complex oncogenic drivers and remarkable biological heterogeneity (Song et al., 2022). OA, as a triterpenoid compound found in plants and herbs, is an important element in human diet (Zhang et al., 2020). It has been used in liver-protecting treatment in clinical practice for several decades (Zhang et al., 2008). In recent years, OA has attracted much attention due to its significant anti-tumor activity and good biosafety, and has gradually become one of the hot molecules in the field of tumor research. However, studies of OA in LUAD have mainly focused on phenotypic observations, while a systematic characterization of its direct molecular targets and downstream signaling networks is still lacking. Here, by integrating network analysis, transcriptomics and multi-omics analyses, we comprehensively delineate the putative targets and mechanisms of action of OA in the experimental context of LUAD. It should be emphasized that while integrated computational workflows are increasingly common, the specific advancement of this study lies not in the methodological pipeline itself, but in its rigorous application to decode the pharmacological profile of OA in LUAD. Distinct from studies that conclude solely with *in silico* findings, this research distinctively contributes an empirical evaluation of dose-dependent target sensitivity. By revealing that TOP2A responds to pharmacologically viable doses while ALOX5AP requires supra-pharmacological concentrations, this study progresses beyond standard computational predictions to offer a critically evaluated, evidence-based foundation for future translational research.

Through PubChem and SwissTargetPrediction, potential target genes for OA were screened. Combined with LUAD-related genes from GeneCards, OMIM, and DisGeNET databases, as well as DEGs from the TCGA-LUAD dataset, the intersection was taken to obtain 21 candidate genes. Subsequently, using two complementary machine learning algorithms, LASSO regression and SVM-RFE, further screening was conducted to obtain 4 core candidate genes. Finally, through the expression difference analysis of the TCGA training set and the GSE140797 testing cohort, as well as the ROC curve assessment, ALOX5AP and TOP2A were ultimately identified as the key biomarkers for OA intervention in LUAD. The results showed that TOP2A was significantly overexpressed in LUAD tumor tissues, whereas ALOX5AP was significantly downregulated. Both genes demonstrated excellent diagnostic value in the TCGA and GEO cohorts.

It is imperative to acknowledge that the initial prioritization of TOP2A and ALOX5AP as core biomarkers heavily relied on statistical discriminative thresholds (i.e., AUC >0.9). However, high diagnostic or discriminative performance does not necessarily translate into actual mechanistic or pharmacological relevance. Therefore, beyond mathematical filtering, the biological plausibility of these targets must be critically evaluated within the context of OA treatment. Biologically, TOP2A is a quintessential nuclear enzyme driving DNA replication and cell cycle progression (Wu et al., 2024). Given that OA is well-documented to inhibit tumor cell proliferation and induce cell cycle arrest (Hu and Qin, 2026), targeting TOP2A provides a highly congruent pharmacological rationale. Similarly, ALOX5AP functions as a critical enzyme in leukotriene synthesis and inflammatory immune regulation (Ye et al., 2021). Since OA is a well-studied triterpenoid with pronounced anti-inflammatory properties, identifying ALOX5AP as a target offers a solid biological foundation for explaining how OA might remodel the inflammatory and immune microenvironment in LUAD. To effectively bridge the gap between pure statistical prioritization and actual pharmacological relevance, the present study employed *in vivo* dose-gradient evaluations. These experiments successfully demonstrated that OA actively modulates these specific targets at the protein level, thereby substantiating their biological responsiveness and pharmacological relevance beyond mere statistical metrics.

The TOP2A gene, located on human chromosome 17 (17q21-22), encodes topoisomerase IIα. This nuclear enzyme regulates DNA topology and cell cycle progression, making it a key marker of cellular proliferation in both normal and tumor tissues (Baiocchi et al., 2016; de Resende et al., 2013). Clinically, TOP2A is a direct target of several classic chemotherapeutic agents, including anthracycline-based drugs (e.g., doxorubicin, epirubicin) and etoposide (Kou et al., 2020). These TOP2A inhibitors are extensively used in treating various malignancies, such as breast and small cell lung cancer (Meng et al., 2022), highlighting the significant translational value of this target. In LUAD, TOP2A is significantly overexpressed. Its expression level is strongly correlated with aggressive tumor features, including enhanced invasiveness, a propensity for distant metastasis, immune evasion, and ultimately, poor patient prognosis (Wu et al., 2024; Wu et al., 2023). Importantly, the prognostic significance of TOP2A remains consistent and statistically significant across multiple independent databases (e.g., GEPIA2, KM Plotter), regardless of whether optimal cutoff values or median expression levels are used for stratification. This consistency firmly establishes TOP2A as a stable and reliable prognostic biomarker in LUAD.

ALOX5AP, located on chromosome 13 (13q12-13), encodes a key protein in the 5-lipoxygenase pathway involved in leukotriene synthesis (Mashima and Okuyama, 2015). This gene demonstrates tumor-specific expression patterns: high expression predicts poor prognosis in acute myeloid leukemia and ovarian cancer, while correlating with favorable outcomes in LUAD (Ye et al., 2021; Li et al., 2025). Notably, ALOX5AP expression shows close associations with tumor immune microenvironment features. In LUAD, its high expression is positively correlated with the tissue-resident memory T cell markers (CD8, CD69, CD103) (Li et al., 2025). In osteosarcoma, it is related to the infiltration of CD8^+^ T cells and the “hot tumor” microenvironment (Chen et al., 2023). Current studies mainly rely on bioinformatics methods and focus on the macroscopic description of the inflammatory-immune axis (Chen et al., 2023; Wang et al., 2021; Xiao and Zhao, 2022). Furthermore, regarding the observed inconsistencies in the prognostic prediction of ALOX5AP across different cutoffs (optimal versus median) and platforms, the optimal cutoff value was adopted as the primary stratification strategy in this study. This approach is widely utilized because gene expression in tumors is characterized by profound molecular heterogeneity and rarely follows a perfect normal distribution; consequently, a strict median cutoff often serves as a subjective and insensitive threshold. However, it must be acknowledged that the loss of statistical significance for ALOX5AP when employing the median cutoff indicates that its prognostic value likely exhibits non-linear characteristics. More importantly, as explicitly evidenced by the newly incorporated multivariate Cox regression model, ALOX5AP failed to retain independent prognostic significance (p = 0.144) when adjusting for major clinical covariates such as age and tumor stage. This indicates that the prognostic relevance of ALOX5AP is highly dependent on specific clinical characteristics within the cohort rather than acting as a standalone driver of survival. Conversely, TOP2A maintained its significant prognostic capacity across optimal cutoffs, median stratifications, and comprehensive multivariate adjustment (HR = 1.14, 95% CI: 1.02–1.274), firmly establishing it as a highly robust, independent prognostic biomarker in LUAD.

The functional mechanism analysis revealed that although TOP2A and ALOX5AP respectively participate in different biological processes, there are potential synergistic regulatory cues between them. TOP2A is mainly enriched in cell cycle-related pathways such as E2F targets and G2M checkpoints, as well as the DNA replication process, which is consistent with its function of promoting tumor cell proliferation. On the other hand, ALOX5AP is significantly enriched in immune inflammatory pathways such as allograft rejection and interferon-γ response, suggesting its potential role in regulating the tumor immune microenvironment. It is noteworthy that both are co-enriched in the “Intestinal immune network for Iga production” pathway, indicating that they may exert a synergistic effect in the mucosal immune response and inflammation regulation of LUAD. Although the expression of TOP2A and ALOX5AP did not show a significant linear correlation in LUAD samples, the GeneMANIA protein interaction network analysis revealed that they share two key adjacent genes: MGST2 and E2F4. Among them, MGST2 catalyzes the generation of LTC4 by combining glutathione (GSH) with LTA4 (an epoxy intermediate produced by the metabolism of arachidonic acid by 5-lipoxygenase) (Thulasingam et al., 2021). It is also an important target for various anti-inflammatory and anti-cancer drugs, which exert their effects by interfering with the biosynthesis of prostaglandins and leukotrienes (Li and Bai, 2025). E2F4, as a well-known transcriptional repressor, is hypothesized to be implicated in cell cycle modulation and has been associated with tumor occurrence and tumor severity (Zou et al., 2022).

Furthermore, the comprehensive genomic and epigenetic analyses provide a critical contextual basis for the pharmacological application of OA. The data revealed that somatic mutation rates (SNV) for TOP2A and ALOX5AP are relatively low, and neither CNV nor methylation statuses exhibit significant correlations with patient survival outcomes. This lack of genomic or epigenetic dominance indicates that the aberrant expression of these targets in LUAD is primarily driven by dynamic transcriptional or post-transcriptional mechanisms, rather than being irreversibly “hard-wired” by DNA alterations (Marine et al., 2020). This observation aligns perfectly with the central pharmacological narrative of this study: because these targets are dynamically regulated, they remain highly susceptible to pharmacological intervention. This fundamental characteristic explains why OA administration can rapidly and effectively downregulate their protein levels *in vivo*, thereby underscoring their viability as dynamic druggable targets rather than fixed, unresponsive genomic biomarkers.

The immune infiltration analysis further suggested the differential regulation of the two on the immune microenvironment of LUAD. ALOX5AP was positively correlated with M2 macrophages with immunosuppressive effects, and negatively correlated with follicular helper T cells. These specific correlations theoretically suggest a potential link to tumor immune escape features. This pattern is consistent with previous reports where ALOX5AP was associated with immunosuppression and M2 macrophage profiles in serous ovarian cancer (Ye et al., 2021). However, these remain bioinformatic associations rather than confirmed functional states in the present LUAD context. Mechanistically integrating these computational immune profiles with the *in vivo* experimental results yields compelling pharmacological hypotheses. The *in silico* analyses demonstrated that high ALOX5AP expression strongly correlates with M2 macrophage polarization and multiple immunosuppressive checkpoints (e.g., PD-L1, TIM-3), indicative of an immune-evasive TME (Ye et al., 2021; Song et al., 2024). Crucially, the *in vivo* data confirmed that high-dose OA administration significantly reduces ALOX5AP protein levels. Therefore, it is hypothesized that the tumor-suppressive effects of OA may be partially mediated by reversing this ALOX5AP-driven immunosuppression, thereby potentially re-sensitizing LUAD to immune surveillance (Tu et al., 2025). Concurrently, the highly sensitive downregulation of TOP2A by low-dose OA may theoretically alter the recruitment patterns of M1 macrophages and activated CD4^+^ T cells (Wu et al., 2024). Although these linkages remain derived from correlative computational predictions, they construct a robust theoretical framework for future investigations evaluating OA as a potential adjuvant to immune checkpoint blockade therapies. TOP2A was positively correlated with M1-type macrophages and activated CD4 memory T cells, suggesting its involvement in the recruitment and activation of immune cells. Similar phenomena have also been observed in hepatocellular carcinoma, where the proportion of CD8 T cells and M1 macrophages was significantly increased in the high TOP2A subgroup (Xie et al., 2025). Moreover, the correlation of ALOX5AP with inhibitory immune checkpoint molecules such as HAVCR2 (TIM-3), CD274 (PD-L1), and PDCD1LG2 (PD-L2) was generally stronger than that of TOP2A, suggesting that ALOX5AP might serve as a theoretical candidate for combined immunotherapy investigations. These findings collectively hypothesize that OA not only exerts anti-tumor properties by inhibiting cell proliferation but also potentially influences the tumor immune microenvironment, demonstrating the multi-mechanism nature of natural compounds in anti-tumor activities.

One of the most intriguing *in silico* observations in this study is the potentially divergent associative patterns of ALOX5AP and TOP2A with TIME metrics. While ALOX5AP demonstrates strong correlations with immunosuppressive features, TOP2A exhibits positive associations with certain activated immune cell subsets. This raises an interesting hypothesis: the dual modulation by OA might theoretically exert a synergistic, bidirectional remodeling effect on the tumor immune microenvironment. However, it must be explicitly noted that this observation is based on indirect correlative patterns. The absence of direct experimental validation regarding immune modulation limits the definitive strength of this conclusion. Consequently, this “opposing roles” concept must be strictly regarded as a computational pharmacology-derived hypothesis, which requires rigorous evaluation through functional *in vivo* immune assays in future studies.

Although specific upstream regulatory networks were prioritized in this study—specifically, the TF-mRNA regulatory axes involving SRF, NFIC, and GATA2 associated with TOP2A and ALOX5AP—it is widely recognized that computational network construction alone does not establish a definitive mechanistic understanding. Because these key upstream regulatory axes have not yet been functionally validated through direct experimental methods, such as chromatin immunoprecipitation (ChIP) or luciferase reporter assays, they must be strictly regarded as a prioritized theoretical framework for future functional exploration, rather than confirmed mechanistic pathways. Despite this limitation, the differential expression analysis revealed that all three transcription factors exhibit a significantly downregulated expression pattern. Although these three transcription factors have no significant correlation with the OS of LUAD patients, they all show a significantly downregulated expression pattern in tumor tissues. Among them, the serum response factor (SRF) is a multifunctional transcriptional factor that is traditionally associated with the modulation of key biological processes such as cell proliferation, migration, cytoskeleton organization, and myogenesis (Costa et al., 2022). NFIC, as a member of the NFI family, has been reported to potentially influence the malignant phenotype of non-small cell lung cancer by mediating m6A mRNA modification (Shi et al., 2024). GATA2 shows a low expression characteristic in LUAD tissues, and its high expression is associated with a significant improvement in OS rate and progression-free survival rate of patients, having important prognostic value (Gong et al., 2021). Furthermore, while molecular docking simulations provided theoretical binding affinities suggesting potential interactions between OA and both TOP2A and ALOX5AP, it is essential to emphasize that these *in silico* models cannot confirm actual biological physical interactions or functional inhibition *in vivo*.

One of the most intriguing findings from the *in vivo* evaluations is the distinctly divergent dose-response dynamics exhibited by TOP2A and ALOX5AP. At the lowest tested dose (37.5 mg/kg, equivalent to 82.1 μmol/kg), TOP2A protein levels were significantly downregulated, with no substantial further reduction observed at higher doses. Given that TOP2A is a quintessential nuclear enzyme essential for DNA replication (Wang et al., 2022), this profound sensitivity and saturation effect imply that TOP2A likely acts as a primary, early-response target of OA at plausible physiological concentrations. Conversely, the suppression of ALOX5AP expression was exclusively observed at the maximum administered dose (150 mg/kg, equivalent to 328.4 μmol/kg). It must be explicitly acknowledged that this represents a supra-pharmacological concentration. This differential dynamic suggests two potential mechanistic interpretations: first, the modulation of ALOX5AP may require a significantly higher pharmacological threshold due to the robust compensatory signaling networks inherent to lipid metabolism and inflammatory pathways (Tufail et al., 2024); alternatively, considering the extreme dosage, its downregulation may likely reflect non-specific toxicity, metabolic stress, or a secondary consequence of late-stage tumor microenvironment remodeling, rather than a true, targeted pharmacological inhibition. Together, these distinct profiles construct a hierarchical pharmacological hypothesis: while OA exhibits dual regulatory capacities, its pharmacological hierarchy positions TOP2A significantly closer to the primary point of molecular intervention. Consequently, TOP2A holds considerably greater validity and potential for future clinical translation compared to the secondary, high-dose-dependent modulation of ALOX5AP. The evaluation of clinical samples suggested the expression characteristics of these two genes in LUAD patients, providing preliminary supportive evidence for their potential clinical association. We further recognize a fundamental limitation inherent in computational network analyses: such *in silico* methodologies inevitably generate a vast volume of correlative data regardless of biological feasibility. As critically pointed out in recent pharmacological discourse, this abundance of computational output can easily lead to over-interpretation or the selective reporting of results (“cherry-picking”) that seamlessly align with preconceived hypotheses. To circumvent this profound caveat, the present study deliberately abandoned the presentation of highly complex, dense global interaction networks, which often provide limited relevance for well-studied metabolites like OA. Instead, the network presentation was strictly streamlined into a simplified schematic focused solely on prioritized theoretical upstream regulatory axes (e.g., the shared TFs SRF, NFIC, and GATA2). Consequently, we explicitly refrain from drawing mechanistic conclusions from these dense interaction networks, acknowledging that they serve merely as an exploratory visual framework rather than confirmed biological evidence.

Crucially, the identification of TOP2A and ALOX5AP in the present study highly corroborates previously reported phenotypic observations regarding OA, effectively reconciling these novel bioinformatic claims with existing literature. First, regarding the finding that OA significantly downregulates TOP2A—a critical enzyme for DNA replication and G2/M checkpoint transition—multiple previous *in vitro* studies have consistently demonstrated that OA induces G2/M cell cycle arrest and apoptosis across various cancers, including lung cancer (Zhao et al., 2015). The current results explicitly reconcile these historical data by pinpointing a precise upstream molecular target. The rapid downregulation of TOP2A by OA provides a direct, specific pharmacological rationale for the downstream phenotypic cell cycle arrest observed by other investigators. Second, concerning the association between ALOX5AP and immune modulation, existing pharmacological literature extensively profiles OA as a potent anti-inflammatory agent, frequently noting its ability to broadly regulate signaling pathways (such as NF-κB or STAT3) to alter macrophage polarization (Ayeleso et al., 2017). The theoretical prediction generated herein—that OA might modulate the tumor immune microenvironment by downregulating the leukotriene regulator ALOX5AP—perfectly aligns with this established theoretical framework (Ye et al., 2021; Song et al., 2024). This suggests that ALOX5AP represents a specific, previously overlooked target axis through which OA potentially exerts its well-documented broad-spectrum anti-inflammatory properties and its hypothesized suppression of M2 macrophages. Therefore, rather than contradicting existing literature, the integrated multi-omics approach utilized in this study successfully bridges the significant gap between the broadly recognized phenotypic effects of OA and its specific, targetable molecular modulators.

To translate these molecular findings into evidence-based clinical practice, the differential dose-dependent responses of the identified targets provide crucial therapeutic insights. The observation that TOP2A is significantly downregulated at lower doses suggests it acts as an early sensitive target, whereas the modulation of ALOX5AP requires higher doses. This distinct target-specific response lays an important evidence-based foundation for designing novel combination therapies. For instance, low-dose OA could potentially be combined with traditional chemotherapeutics targeting TOP2A (Ayeleso et al., 2017), while higher doses might be necessary when synergizing with immune checkpoint inhibitors targeting the ALOX5AP/M2 macrophage pathway (Gao et al., 2022). However, the current animal models possess inherent limitations. As previously mentioned, the *in vivo* dosages function primarily as mechanistic probes to evaluate target engagement. Future translational studies must systematically address the pharmacokinetics, bioavailability, and precise dosing regimens of OA to facilitate its transition from a preclinical candidate to a viable clinical therapeutic agent (Lee et al., 2024).

Despite the integrated approach employed, several critical limitations must be acknowledged. First, the *in silico* network analysis fundamentally relies on interlinked public databases (e.g., SwissTargetPrediction, GeneCards), which poses inherent systemic caveats. Cross-platform information sharing can create an “echo chamber” effect, amplifying poorly curated data and untested assumptions based merely on structural similarity. As recently highlighted by Diao et al. (Diao et al., 2026), these repositories are prone to historical false positives driven by excessive dosing or pan-assay interfering substances (PAINS). This is particularly critical because OA, a natural pentacyclic triterpene, shares structural characteristics with known PAINS. Consequently, the initial computational predictions may inherently include false positives rather than true specific ligand-receptor interactions. Although we partially mitigated this bias by cross-referencing with real transcriptomic data (TCGA DEGs), the *in silico* findings must be strictly regarded as preliminary hypothesis-generating tools. However, while relying on multiple databases inevitably carries the risk of initially incorporating low-confidence associations, this potential bias was significantly mitigated in the present study. The application of a strict intersection strategy, followed by dual machine learning algorithms (LASSO and SVM-RFE) and real transcriptomic cross-validation, acted as stringent computational filters, effectively minimizing early-stage false positives. Second, the majority of the current findings provide associative evidence rather than establishing direct causal mechanistic links. For instance, the extensive immune infiltration analyses (e.g., CIBERSORT, ssGSEA) are fundamentally correlative, and interpretations regarding theoretical immune suppression or M2 macrophage polarization lack support from direct functional immune assays (such as *in vitro* co-culture models). Furthermore, while the *in vivo* xenograft models successfully demonstrated a phenotypic reduction in tumor volume and downstream pharmacological modulation of TOP2A and ALOX5AP, the experimental design lacks deep mechanistic endpoints (such as *in vivo* flow cytometry or cell cycle markers like Ki-67) and upstream pathway-level validations (e.g., SRF or NFIC). The current data do not confirm direct biophysical interactions; future investigations utilizing direct binding assays (e.g., surface plasmon resonance) and loss/gain-of-function models (e.g., CRISPR/Cas9) are essential to unequivocally elucidate causality. Third, the generalizability of the current findings is restricted by the experimental and clinical models employed. The *in vivo* evaluation relied on a single LUAD cell line (A549), failing to capture the profound molecular heterogeneity (e.g., KRAS, EGFR mutational landscapes) characteristic of the disease. Similarly, the preliminary assessment of clinical samples was based on a small cohort (n = 20), which inherently limits statistical power and cannot adequately adjust for potential confounders such as smoking history, gender, and underlying comorbidities. Furthermore, while the machine learning pipeline achieved highly discriminative AUC values (>0.9) in the external validation cohort (GSE140797), it must be strictly acknowledged that this dataset contains an extremely small sample size (n = 7 pairs). As highlighted by recent methodological standards, high AUC values derived from such small cohorts carry a significant risk of being overestimated due to mathematical overfitting. Consequently, these *in silico* diagnostic metrics should be cautiously interpreted as preliminary indicators rather than definitive clinical parameters. To partially alleviate this computational limitation, the expression patterns were independently assessed via RT-qPCR in the aforementioned in-house clinical cohort (n = 20), which successfully corroborated the *in silico* trends, though large-scale validation remains necessary. Future studies must incorporate diverse LUAD cell panels, patient-derived xenografts (PDXs), and large-scale, comprehensively annotated multi-center clinical cohorts to rigorously ascertain the true prognostic value and universality of these targets. Finally, bridging these molecular discoveries to real-world clinical applications faces substantial translational challenges due to the inherent pharmacokinetic (PK) limitations of OA. As a pentacyclic triterpenoid, OA exhibits notoriously poor aqueous solubility and low oral bioavailability. Consequently, the high *in vivo* doses (up to 150 mg/kg, equivalent to 328.4 μmol/kg) required to achieve observable tumor suppression in this study represent supra-pharmacological concentrations and cannot be linearly extrapolated to feasible human clinical regimens. Attaining effective intratumoral concentrations in patients using unformulated OA would necessitate clinically impractical doses. Therefore, future translational research must prioritize strategies to enhance the PK profile of OA, such as developing advanced nano-delivery systems or synthesizing highly bioavailable structural analogues, before realistic clinical trials can be initiated.

In conclusion, this study employed an integrated computational approach as a preliminary starting point to investigate the pharmacological associations of OA in LUAD. Comprehensive *in silico* screening and theoretical binding assessments suggest that TOP2A and ALOX5AP are potential pharmacological biomarkers associated with OA treatment, rather than definitively confirmed specific targets. Subsequent *in vivo* and preliminary clinical evaluations demonstrated significant pharmacological associations, revealing that OA administration downregulates these biomarkers, which are implicated in cell cycle regulation and immune-inflammatory pathways. However, it must be explicitly emphasized that the current multi-omics and experimental data primarily establish correlative rather than causal relationships. While these findings construct a systematic theoretical framework for understanding the pharmacological effects of OA, future functional investigations—such as loss-of-function and gain-of-function experiments—are fundamentally required. Such rigorous studies are essential to unequivocally establish causality, rule out non-specific compound interferences, and validate the specific roles of these targets. Ultimately, this work provides a robust pharmacological and theoretical foundation for further exploring the therapeutic potential of OA.

## Data Availability

The raw data supporting the conclusions of this article will be made available by the authors, without undue reservation.

## Supplementary material

SUPPLEMENTARY FIGURE S1 OA structure and pathway enrichment. **(A)** OA structure. **(B,C)** Top 20 enriched pathways of drug targets, **(B)** color-coded by category; **(C)** bar length and color ranked by p value.

SUPPLEMENTARY FIGURE S2 Prognostic value of ALOX5AP and TOP2A in LUAD. **(A)** Prognostic analysis of ALOX5AP and TOP2A in the TCGA-LUAD cohort. **(B,C)** Independent validation in the GEPIA2 **(B)** and Kaplan-Meier Plotter **(C)** databases. Patients were stratified into high- and low-expression groups based on the median expression level of each gene as the cutoff.

SUPPLEMENTARY FIGURE S3 Prognostic value of ALOX5AP and TOP2A in LUAD. **(A)** TOP2A SNV predict shorter DFI and PFS. **(B)** Correlation analysis of ALOX5AP and TOP2A CNV levels with the four survival endpoints. **(C)** Correlation analysis of ALOX5AP and TOP2A methylation levels with the four survival endpoints. Amp, Amplification; Dele, Deletion; WT, Wild Type.

SUPPLEMENTARY FIGURE S4 Immune Infiltration and Immune Checkpoint Correlation Analysis of ALOX5AP and TOP2A in LUAD. **(A)** CIBERSORT analysis. **(B)** ssGSEA analysis. **(C)** ESTIMATE algorithm. **(D)** Checkpoint analysis.

SUPPLEMENTARY FIGURE S5 Biomarker - pathway - common target network. Red nodes represent biomarkers, green nodes represent pathways, and blue nodes represent target genes.

SUPPLEMENTARY FIGURE S6 Transcription factor-related analyses. **(A)** Correlation between ALOX5AP/TOP2A and transcription factors (SRF, NFIC, GATA2) expression. **(B)** Correlation analysis of ALOX5AP and TOP2A expression in LUAD. **(C)** Expression of three transcription factors in unpaired and paired TCGA-LUAD tumor-normal samples. **(D)** Prognostic analysis (overall survival) of the three transcription factors in the TCGA-LUAD cohort. R, correlation coefficient; ns, not statistically different, *p <0.05, **p<0.01, ***p<0.001, ****p<0.0001.

SUPPLEMENTARY FIGURE S7 Molecular docking simulations of established reference ligands (positive controls) with ALOX5AP and TOP2A. **(A)** 3D binding mode of ALOX5AP with MK-886 (binding energy: -9.7 kcal/mol). **(B)** 3D binding mode of ALOX5AP with Veliflapon (binding energy: -10.3 kcal/mol). **(C)** 3D binding mode of TOP2A with Doxorubicin (binding energy: -10.1 kcal/mol). **(D)** 3D binding mode of TOP2A with Etoposide (binding energy: -9.4 kcal/mol).

SUPPLEMENTARY FIGURE S8 Dynamic stability analysis of TOP2A-OA and ALOX5AP-OA complexes from 100 ns MD simulations. **(A)** RMSD curves; **(B)** RMSF profiles of protein residues; **(C)** total energy fluctuations; **(D)** time-dependent hydrogen bond counts between OA and target proteins.
