# Supplementary material for: Integrating network analysis and machine learning to explore the pharmacological targets and associations of oleanolic acid in lung adenocarcinoma
Source: Front Pharmacol. 2026 Sep 18;17:1841232. doi: 10.3389/fphar.2026.1841232 (PMC13630726; doi:10.3389/fphar.2026.1841232)
Supplement: Supplementary file 1 [file Table1.docx]

Suppl_Table1. List of 100 oleanolic acid target genes.

| Target | Common name | Uniprot ID | ChEMBL ID | Target Class | Probability* |
| --- | --- | --- | --- | --- | --- |
| Protein-tyrosine phosphatase 1B | PTPN1 | P18031 | CHEMBL335 | Phosphatase | 0.971605984 |
| DNA polymerase beta | POLB | P06746 | CHEMBL2392 | Enzyme | 0.762670917 |
| Aldo-keto reductase family 1 member B10 | AKR1B10 | O60218 | CHEMBL5983 | Enzyme | 0.737539094 |
| Nuclear receptor ROR-gamma | RORC | P51449 | CHEMBL1741186 | Nuclear receptor | 0.662093991 |
| Receptor-type tyrosine-protein phosphatase F (LAR) | PTPRF | P10586 | CHEMBL3521 | Membrane receptor | 0.662093991 |
| T-cell protein-tyrosine phosphatase | PTPN2 | P17706 | CHEMBL3807 | Phosphatase | 0.662093991 |
| 11-beta-hydroxysteroid dehydrogenase 1 | HSD11B1 | P28845 | CHEMBL4235 | Enzyme | 0.662093991 |
| Low molecular weight phosphotyrosine protein phosphatase | ACP1 | P24666 | CHEMBL4903 | Phosphatase | 0.662093991 |
| Phosphodiesterase 4D | PDE4D | Q08499 | CHEMBL288 | Phosphodiesterase | 0.611730742 |
| Dual specificity phosphatase Cdc25B | CDC25B | P30305 | CHEMBL4804 | Phosphatase | 0.586636873 |
| Phospholipase A2 group 1B | PLA2G1B | P04054 | CHEMBL4426 | Enzyme | 0.569892605 |
| CD81 antigen | CD81 | P60033 | CHEMBL1075180 | Surface antigen | 0.528015714 |
| Prostaglandin E synthase | PTGES | O14684 | CHEMBL5658 | Enzyme | 0.184423157 |
| Fatty acid binding protein muscle | FABP3 | P05413 | CHEMBL3344 | Fatty acid binding protein family | 0.109013468 |
| Fatty acid binding protein epidermal | FABP5 | Q01469 | CHEMBL3674 | Fatty acid binding protein family | 0.109013468 |
| Fatty acid-binding protein, liver | FABP1 | P07148 | CHEMBL5421 | Fatty acid binding protein family | 0.109013468 |
| Telomerase reverse transcriptase | TERT | O14746 | CHEMBL2916 | Enzyme | 0.109013468 |
| Dual specificity phosphatase Cdc25A | CDC25A | P30304 | CHEMBL3775 | Phosphatase | 0.109013468 |
| Nitric oxide synthase, inducible | NOS2 | P35228 | CHEMBL4481 | Enzyme | 0.109013468 |
| Protein-tyrosine phosphatase 1C | PTPN6 | P29350 | CHEMBL3166 | Phosphatase | 0.109013468 |
| Acyl-CoA desaturase | SCD | O00767 | CHEMBL5555 | Enzyme | 0.109013468 |
| Peroxisome proliferator-activated receptor gamma | PPARG | P37231 | CHEMBL235 | Nuclear receptor | 0.100634432 |
| Cyclooxygenase-2 | PTGS2 | P35354 | CHEMBL230 | Oxidoreductase | 0.100634432 |
| Prolyl endopeptidase | PREP | P48147 | CHEMBL3202 | Protease | 0.100634432 |
| Protein farnesyltransferase | FNTA FNTB | P49354 P49356 | CHEMBL2094108 | Enzyme | 0.100634432 |
| Cytochrome P450 19A1 | CYP19A1 | P11511 | CHEMBL1978 | Cytochrome P450 | 0.100634432 |
| Leukotriene B4 receptor 1 | LTB4R | Q15722 | CHEMBL3911 | Family A G protein-coupled receptor | 0.100634432 |
| Mineralocorticoid receptor | NR3C2 | P08235 | CHEMBL1994 | Nuclear receptor | 0.100634432 |
| Peroxisome proliferator-activated receptor alpha | PPARA | Q07869 | CHEMBL239 | Nuclear receptor | 0.100634432 |
| Androgen Receptor | AR | P10275 | CHEMBL1871 | Nuclear receptor | 0.100634432 |
| Fatty acid binding protein adipocyte | FABP4 | P15090 | CHEMBL2083 | Fatty acid binding protein family | 0.100634432 |
| Protein-tyrosine phosphatase 2C | PTPN11 | Q06124 | CHEMBL3864 | Phosphatase | 0.100634432 |
| Corticosteroid binding globulin | SERPINA6 | P08185 | CHEMBL2421 | Secreted protein | 0.100634432 |
| Testis-specific androgen-binding protein | SHBG | P04278 | CHEMBL3305 | Secreted protein | 0.100634432 |
| Glucose-6-phosphate 1-dehydrogenase | G6PD | P11413 | CHEMBL5347 | Enzyme | 0.100634432 |
| Cytochrome P450 51 (by homology) | CYP51A1 | Q16850 | CHEMBL3849 | Cytochrome P450 | 0.100634432 |
| Peroxisome proliferator-activated receptor delta | PPARD | Q03181 | CHEMBL3979 | Nuclear receptor | 0.100634432 |
| Glucocorticoid receptor | NR3C1 | P04150 | CHEMBL2034 | Nuclear receptor | 0.100634432 |
| Cyclooxygenase-1 | PTGS1 | P23219 | CHEMBL221 | Oxidoreductase | 0.100634432 |
| Anandamide amidohydrolase | FAAH | O00519 | CHEMBL2243 | Enzyme | 0.100634432 |
| Progesterone receptor | PGR | P06401 | CHEMBL208 | Nuclear receptor | 0.100634432 |
| Carboxylesterase 2 | CES2 | O00748 | CHEMBL3180 | Enzyme | 0.100634432 |
| 11-beta-hydroxysteroid dehydrogenase 2 | HSD11B2 | P80365 | CHEMBL3746 | Enzyme | 0.100634432 |
| Niemann-Pick C1-like protein 1 | NPC1L1 | Q9UHC9 | CHEMBL2027 | Other membrane protein | 0.100634432 |
| Sigma opioid receptor | SIGMAR1 | Q99720 | CHEMBL287 | Membrane receptor | 0.100634432 |
| Cytochrome P450 17A1 | CYP17A1 | P05093 | CHEMBL3522 | Cytochrome P450 | 0.100634432 |
| Estrogen receptor beta | ESR2 | Q92731 | CHEMBL242 | Nuclear receptor | 0.100634432 |
| Prostanoid EP2 receptor (by homology) | PTGER2 | P43116 | CHEMBL1881 | Family A G protein-coupled receptor | 0.100634432 |
| HMG-CoA reductase | HMGCR | P04035 | CHEMBL402 | Oxidoreductase | 0.100634432 |
| LXR-alpha | NR1H3 | Q13133 | CHEMBL2808 | Nuclear receptor | 0.100634432 |
| Beta-secretase 1 | BACE1 | P56817 | CHEMBL4822 | Protease | 0.100634432 |
| SUMO-activating enzyme | SAE1 UBA2 | Q9UBE0 Q9UBT2 | CHEMBL2095174 | Enzyme | 0.100634432 |
| Serotonin transporter | SLC6A4 | P31645 | CHEMBL228 | Electrochemical transporter | 0.100634432 |
| Cytochrome P450 2C19 | CYP2C19 | P33261 | CHEMBL3622 | Cytochrome P450 | 0.100634432 |
| DNA topoisomerase I | TOP1 | P11387 | CHEMBL1781 | Isomerase | 0.100634432 |
| Protein kinase C eta | PRKCH | P24723 | CHEMBL3616 | Kinase | 0.100634432 |
| Prostanoid EP4 receptor (by homology) | PTGER4 | P35408 | CHEMBL1836 | Family A G protein-coupled receptor | 0.100634432 |
| Arachidonate 5-lipoxygenase | ALOX5 | P09917 | CHEMBL215 | Oxidoreductase | 0.100634432 |
| Muscarinic acetylcholine receptor M2 | CHRM2 | P08172 | CHEMBL211 | Family A G protein-coupled receptor | 0.100634432 |
| Glutamate receptor ionotropic kainate 1 | GRIK1 | P39086 | CHEMBL1918 | Ligand-gated ion channel | 0.100634432 |
| Glutamate receptor ionotropic kainate 2 | GRIK2 | Q13002 | CHEMBL3683 | Ligand-gated ion channel | 0.100634432 |
| p53-binding protein Mdm-2 | MDM2 | Q00987 | CHEMBL5023 | Other nuclear protein | 0.100634432 |
| Type-1 angiotensin II receptor | AGTR1 | P30556 | CHEMBL227 | Family A G protein-coupled receptor | 0.100634432 |
| Nuclear receptor ROR-alpha | RORA | P35398 | CHEMBL5868 | Nuclear receptor | 0.100634432 |
| Prostanoid IP receptor | PTGIR | P43119 | CHEMBL1995 | Family A G protein-coupled receptor | 0.100634432 |
| Bile acid receptor FXR | NR1H4 | Q96RI1 | CHEMBL2047 | Nuclear receptor | 0.100634432 |
| DNA topoisomerase II alpha | TOP2A | P11388 | CHEMBL1806 | Isomerase | 0.100634432 |
| Nuclear receptor subfamily 1 group I member 3 (by homology) | NR1I3 | Q14994 | CHEMBL5503 | Nuclear receptor | 0.100634432 |
| Dopamine transporter | SLC6A3 | Q01959 | CHEMBL238 | Electrochemical transporter | 0.100634432 |
| Adenosine A3 receptor | ADORA3 | P0DMS8 | CHEMBL256 | Family A G protein-coupled receptor | 0.100634432 |
| Free fatty acid receptor 1 | FFAR1 | O14842 | CHEMBL4422 | Family A G protein-coupled receptor | 0.100634432 |
| MAP kinase ERK1 | MAPK3 | P27361 | CHEMBL3385 | Kinase | 0.100634432 |
| Cannabinoid receptor 1 | CNR1 | P21554 | CHEMBL218 | Family A G protein-coupled receptor | 0.100634432 |
| Acyl coenzyme A:cholesterol acyltransferase | CES1 | P23141 | CHEMBL2265 | Enzyme | 0.100634432 |
| G-protein coupled bile acid receptor 1 | GPBAR1 | Q8TDU6 | CHEMBL5409 | Family A G protein-coupled receptor | 0.100634432 |
| Prostanoid EP1 receptor | PTGER1 | P34995 | CHEMBL1811 | Family A G protein-coupled receptor | 0.100634432 |
| Vanilloid receptor | TRPV1 | Q8NER1 | CHEMBL4794 | Voltage-gated ion channel | 0 |
| Heat shock factor protein 1 | HSF1 | Q00613 | CHEMBL5869 | Other cytosolic protein | 0 |
| Solute carrier family 22 member 6 (by homology) | SLC22A6 | Q4U2R8 | CHEMBL1641347 | Electrochemical transporter | 0 |
| G protein-coupled receptor 44 | PTGDR2 | Q9Y5Y4 | CHEMBL5071 | Family A G protein-coupled receptor | 0 |
| Estrogen receptor alpha | ESR1 | P03372 | CHEMBL206 | Nuclear receptor | 0 |
| Squalene synthetase (by homology) | FDFT1 | P37268 | CHEMBL3338 | Enzyme | 0 |
| Interleukin-6 | IL6 | P05231 | CHEMBL1795129 | Secreted protein | 0 |
| Glutamine synthetase | GLUL | P15104 | CHEMBL4612 | Ligase | 0 |
| Cysteinyl leukotriene receptor 1 | CYSLTR1 | Q9Y271 | CHEMBL1798 | Family A G protein-coupled receptor | 0 |
| Matrix metalloproteinase 3 | MMP3 | P08254 | CHEMBL283 | Protease | 0 |
| Matrix metalloproteinase 1 | MMP1 | P03956 | CHEMBL332 | Protease | 0 |
| Matrix metalloproteinase 2 | MMP2 | P08253 | CHEMBL333 | Protease | 0 |
| Sphingosine 1-phosphate receptor Edg-5 | S1PR2 | O95136 | CHEMBL2955 | Family A G protein-coupled receptor | 0 |
| Indoleamine 2,3-dioxygenase | IDO1 | P14902 | CHEMBL4685 | Enzyme | 0 |
| Butyrylcholinesterase | BCHE | P06276 | CHEMBL1914 | Hydrolase | 0 |
| Steroid 5-alpha-reductase 2 | SRD5A2 | P31213 | CHEMBL1856 | Oxidoreductase | 0 |
| Thyroid hormone receptor alpha | THRA | P10827 | CHEMBL1860 | Nuclear receptor | 0 |
| Thyroid hormone receptor beta-1 | THRB | P10828 | CHEMBL1947 | Nuclear receptor | 0 |
| AMP deaminase 2 | AMPD2 | Q01433 | CHEMBL2997 | Enzyme | 0 |
| 5-lipoxygenase activating protein | ALOX5AP | P20292 | CHEMBL4550 | Other cytosolic protein | 0 |
| Endothelin receptor ET-B | EDNRB | P24530 | CHEMBL1785 | Family A G protein-coupled receptor | 0 |
| Endothelin receptor ET-A | EDNRA | P25101 | CHEMBL252 | Family A G protein-coupled receptor | 0 |
| Leukocyte adhesion glycoprotein LFA-1 alpha | ITGAL | P20701 | CHEMBL1803 | Adhesion | 0 |
| Cholecystokinin B receptor | CCKBR | P32239 | CHEMBL298 | Family A G protein-coupled receptor | 0 |
