# Supplementary material for: Integrating network analysis and machine learning to explore the pharmacological targets and associations of oleanolic acid in lung adenocarcinoma
Source: Front Pharmacol. 2026 Sep 18;17:1841232. doi: 10.3389/fphar.2026.1841232 (PMC13630726; doi:10.3389/fphar.2026.1841232)
Supplement: Supplementary file 2 [file Table2.docx]

**Suppl_Fig1**. OA structure and pathway enrichment. (A) OA structure. (B-C) Top 20 enriched pathways of drug targets, (B) color-coded by category; (C) bar length and color ranked by p value.

**Suppl_Fig2**. Prognostic value of ALOX5AP and TOP2A in LUAD. (A) Prognostic analysis of ALOX5AP and TOP2A in the TCGA-LUAD cohort. (B-C) Independent validation in the GEPIA2 (B) and Kaplan-Meier Plotter (C) databases. Patients were stratified into high- and low-expression groups based on the median expression level of each gene as the cutoff.

**Suppl_Fig3**. Prognostic value of ALOX5AP and TOP2A in LUAD. (A) TOP2A SNV predict shorter DFI and PFS. (B) Correlation analysis of ALOX5AP and TOP2A CNV levels with the four survival endpoints. (C) Correlation analysis of ALOX5AP and TOP2A methylation levels with the four survival endpoints. Amp, Amplification; Dele, Deletion; WT, Wild Type.

**Suppl_Fig4**. Immune Infiltration and Immune Checkpoint Correlation Analysis of ALOX5AP and TOP2A in LUAD. (A) CIBERSORT analysis. (B) ssGSEA analysis. (C) ESTIMATE algorithm. (D) Checkpoint analysis.

**Suppl_Fig5**. Biomarker - pathway - common target network. Red nodes represent biomarkers, green nodes represent pathways, and blue nodes represent target genes.

**Suppl_Fig6**. Transcription factor-related analyses. (A) Correlation between ALOX5AP/TOP2A and transcription factors (SRF, NFIC, GATA2) expression. (B) Correlation analysis of ALOX5AP and TOP2A expression in LUAD. (C) Expression of three transcription factors in unpaired and paired TCGA-LUAD tumor-normal samples. (D) Prognostic analysis (overall survival) of the three transcription factors in the TCGA-LUAD cohort. R, correlation coefficient; ns, not statistically different, *p <0.05, **p<0.01, ***p<0.001, ****p<0.0001.

**Suppl_Fig7**. Molecular docking simulations of established reference ligands (positive controls) with ALOX5AP and TOP2A. (A) 3D binding mode of ALOX5AP with MK-886 (binding energy: -9.7 kcal/mol). (B) 3D binding mode of ALOX5AP with Veliflapon (binding energy: -10.3 kcal/mol). (C) 3D binding mode of TOP2A with Doxorubicin (binding energy: -10.1 kcal/mol). (D) 3D binding mode of TOP2A with Etoposide (binding energy: -9.4 kcal/mol).

**Suppl_Fig8**. Dynamic stability analysis of TOP2A-OA and ALOX5AP-OA complexes from 100 ns MD simulations. (A) RMSD curves; (B) RMSF profiles of protein residues; (C) total energy fluctuations; (D) time-dependent hydrogen bond counts between OA and target proteins.
